# Chemically Synthesized, Self‐Assembling Small Interfering RNA‐Prohead RNA Molecules Trigger Dicer‐Independent Gene Silencing

**DOI:** 10.1002/chem.202103995

**Published:** 2022-01-12

**Authors:** Alyssa C. Hill, Daniël van Leeuwen, Verena Schlösser, Alok Behera, Bogdan Mateescu, Jonathan Hall

**Affiliations:** ^1^ ETH Zürich Department of Chemistry and Applied Biosciences Institute of Pharmaceutical Sciences Vladimir-Prelog-Weg 1–5/10 8093 Zürich Switzerland; ^2^ ETH Zürich Department of Biology Wolfgang-Pauli-Strasse 27 8093 Zürich Switzerland

**Keywords:** packaging RNA, prohead RNA, RNA nanotechnology, small interfering RNA, three-way junction

## Abstract

RNA interference (RNAi) mediated by small interfering RNA (siRNA) duplexes is a powerful therapeutic modality, but the translation of siRNAs from the bench into clinical application has been hampered by inefficient delivery in vivo. An innovative delivery strategy involves fusing siRNAs to a three‐way junction (3WJ) motif derived from the phi29 bacteriophage prohead RNA (pRNA). Chimeric siRNA‐3WJ molecules are presumed to enter the RNAi pathway through Dicer cleavage. Here, we fused siRNAs to the phi29 3WJ and two phylogenetically related 3WJs. We confirmed that the siRNA‐3WJs are substrates for Dicer in vitro. However, our results reveal that siRNA‐3WJs transfected into Dicer‐deficient cell lines trigger potent gene silencing. Interestingly, siRNA‐3WJs transfected into an Argonaute 2‐deficient cell line also retain some gene silencing activity. siRNA‐3WJs are most efficient when the antisense strand of the siRNA duplex is positioned 5′ of the 3WJ (5′‐siRNA‐3WJ) relative to 3′ of the 3WJ (3′‐siRNA‐3WJ). This work sheds light on the functional properties of siRNA‐3WJs and offers a design rule for maximizing their potency in the human RNAi pathway.

## Graphical abstract

**Chimeric molecules** fusing small interfering RNA (siRNA) duplexes to prohead RNA (pRNA) three‐way junctions (3WJs) were designed. The siRNA‐3WJs self‐assembled in vitro. siRNA‐3WJs transfected into Dicer‐deficient cell lines triggered potent gene silencing. siRNA‐3WJs are most efficient when the antisense strand of the siRNA duplex is positioned 5′ of the 3WJ (5′‐siRNA‐3WJ) relative to 3′ of the 3WJ (3′‐siRNA‐3WJ).

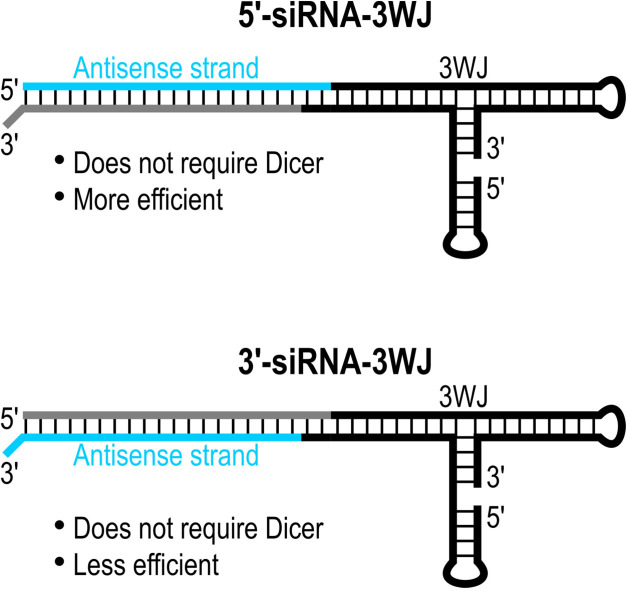

Small interfering RNAs (siRNAs) with 19 base pair (bp) duplexes and dinucleotide 3′ overhangs are the canonical exogenous triggers of RNA interference (RNAi).^[1]^ In the cytoplasm, siRNAs associate with one of four Argonaute proteins (Ago1–4) in the RNA‐induced silencing complex (RISC): Ago1, Ago3, and Ago4 silence gene expression through translational repression and deadenylation, while Ago2 drives the catalytic cleavage of messenger RNA (mRNA) sequences that are complementary to the antisense strand of the siRNA duplex.^[2]^

Although RNAi is a powerful therapeutic modality, degradation in serum, clearance from circulation, and inefficient uptake by target cells have hampered the translation of siRNAs from bench to bedside.^[3]^ Today, only three siRNA drugs are approved for clinical use in the US and EU.^[4]^ Conjugating siRNAs to *N*‐acetylgalactosamine (GalNAc) achieves delivery to hepatocytes and constitutes a major breakthrough in the oligonucleotide therapeutics field.^[5]^ Nevertheless, the development of new, effective technologies is required to deliver systemically administered siRNAs to tissues beyond the liver.^[6]^

RNA itself is a promising platform for siRNA delivery.^[7]^ Owing to canonical Watson‐Crick base pairing (that is, A : U and C : G), RNA is highly programmable and can be manipulated with the simplicity characteristic of DNA.^[8]^ RNA also engages in a variety of non‐canonical interactions, which give rise to complex 3D architectures and a functional repertoire that rivals proteins. Since RNA is chemically, structurally, and functionally modular,^[9]^ RNA motifs can be mixed and matched to yield composite structures with novel properties. For example, grafting siRNAs onto RNA branched motifs,^[10]^ rings,^[11]^ cubes,^[12]^ and tetrahedra^[13]^ can achieve tissue‐specific delivery,[^10a^, ^10b^, ^10c^, ^10d^, ^10e^, ^10g^, ^11b^] enhanced cellular uptake,[^10g^, ^10h^, ^10i^, ^10j^, ^11b^, ^13^] conditional RNAi activation,[^11b^, ^12^] alternative processing by the RNAi machinery,[^10f^, ^10h^, ^10j^] more potent/prolonged RNAi activity,[^10i^, ^10j^, ^10k^, ^11b^, ^13^] and synergistic/combinatorial RNAi.[^10f^, ^10g^, ^10h^, ^10i^, ^10j^, ^10k^, ^11b^, ^12^]

A three‐way junction (3WJ) motif derived from the phi29 bacteriophage prohead RNA (pRNA) is thermodynamically stable^[10a]^ and can host large, functional payloads.^[14]^ *In vivo*, the blood half‐life measured for a 2′‐deoxyfluoro (2′‐F) phi29 3WJ was over 6.5 h, compared with less than 5 min for a 2′‐F siRNA.^[10a]^ Chimeric molecules that fuse siRNAs to the phi29 3WJ can be programmed for tissue‐specific delivery using small molecule ligands[^10a^, ^10b^, ^10c^] or nucleic acid aptamers.[^10d^, ^10e^] Moreover, siRNA‐3WJs silence gene expression as efficiently as their sequence‐matched siRNA counterparts.[^10a^, ^10c^]

siRNA‐3WJs are presumed to enter the RNAi pathway through Dicer cleavage.[^10d^, ^15^] Here, we fused siRNAs to the phi29, M2, and SF5 3WJs and confirmed that the siRNA‐3WJs are substrates for Dicer in vitro. However, our results reveal that siRNA‐3WJs transfected into Dicer‐deficient cell lines trigger potent gene silencing. Interestingly, siRNA‐3WJs transfected into an Ago2‐deficient cell line retain some gene silencing activity, suggesting that siRNA‐3WJs may associate with Ago1, Ago3, or Ago4. siRNA‐3WJs are most efficient when the antisense strand of the siRNA duplex is positioned 5′, as opposed to 3′, of the 3WJ.

pRNA is a noncoding RNA produced by phi29‐like bacteriophages.^[17]^ It forms a multimeric ring on immature viral capsids, or proheads, and gears a DNA packaging motor.^[18]^ Phylogenetically related pRNAs have diverse sequences,^[19]^ but they share a common secondary structure consisting of six helices, several bulge loops, two kissing loops, and a 3WJ.^[20]^ The 3WJ self‐assembles from three RNA oligomers in vitro.^[21]^ Relative to the phi29 3WJ, the M2 and SF5 3WJs have higher thermodynamic stabilities^[22]^ and may be better positioned for siRNA delivery. We designed phi29, M2, and SF5 3WJ constructs using three strands, as described previously.^[22]^ The sequences of strand 1 (S1), strand 2 (S2), and strand 3 (S3) are provided in Table S1. Oligoribonucleotides were produced by solid‐phase synthesis. Mass and purity were verified by liquid chromatography‐mass spectrometry (LC‐MS) (Table S2). Self‐assembly was confirmed by annealing the strands in equimolar concentrations and subjecting them to non‐denaturing polyacrylamide gel electrophoresis (PAGE) (Figure S1).

To assess their thermal stabilities, we subjected the 3WJs to UV melting. Melting curves showed sharp transitions consistent with the cooperative melting of 3WJs into single strands (Figure S2). Melting curves for single strands (Figure S3) and paired strands (Figure S4) did not show transitions that would compete with the 3WJ. Melting temperatures (*T*_m_s) were calculated from first derivative analyses of nonlinear fit melting curves. The 3WJ with the highest thermal stability was M2 (*T*_m_=59.3 °C), followed by SF5 (*T*_m_=55.6 °C) and phi29 (*T*_m_=51.4 °C) (Table 1). To assess their serum stabilities, we incubated the 3WJs in 50 % mouse serum and subjected the samples to non‐denaturing PAGE (Figure S5). Half‐lives (*t*_1/2_s) were calculated by fitting 3WJ intensity data to one‐phase exponential decay functions. The 3WJ with the highest serum stability was M2 (*t*_1/2_=5.8 h), followed by phi29 (*t*_1/2_=1.4 h) and SF5 (*t*_1/2_=0.8 h) (Table 1). The M2 3WJ exhibited the highest thermal and serum stability and therefore was selected as a lead scaffold.

**Table 1:** Thermal and serum stabilities of pRNA 3WJs. *T*_m_, melting temperature; *t*_1/2_, half‐life.

Construct	*T*_m_ [°C]	*t*_1/2_ [h]
Phi29 3WJ	51.4	1.4
M2 3WJ	59.3	5.8
SF5 3WJ	55.6	0.8

Next, we fused an siRNA targeting Renilla luciferase (siREN) or a control siRNA (siRND) to the 3WJs. siRND has six randomized base‐pair positions.^[24]^ The single‐stranded randomized RNAs hybridize to form the most thermodynamically stable (that is, complementary) duplexes^[24]^ and were not expected to interfere with the formation of the siRNA‐3WJ structures. We generated 5′‐siRNA‐3WJs by positioning the antisense strand 5′ of the 3WJ (Figure 1A) and 3′‐siRNA‐3WJs by positioning the antisense strand 3′ of the 3WJ (Figure 1B). We incorporated a dinucleotide adapter into our designs to account for a dinucleotide 3′ overhang on the fused siRNA. Our designs use two strands, chimera 1 (C1) and chimera 2 (C2) (Table S3), following a bipartite approach.^[15a]^ RNAstructure software^[25]^ predicted that the paired strands fold as designed (Figure S6). Oligoribonucleotides were produced and assessed for self‐assembly using the same methods described above (Table S4, Figures 1C and S7). In the non‐denaturing gels, multiple bands observed for single‐stranded RNAs (for example, 5′‐siREN‐M2 C2, 5′‐siREN‐M2 C1, and 3′‐siREN‐M2 C1) were attributed to alternative secondary structures, rather than impurities (UV purity>90 %; Figure S8). Multiple bands observed for double‐stranded RNAs were attributed to excluded single strands, alternative secondary structures, and/or alternative conformations of the same secondary structure.

**Figure 1:**
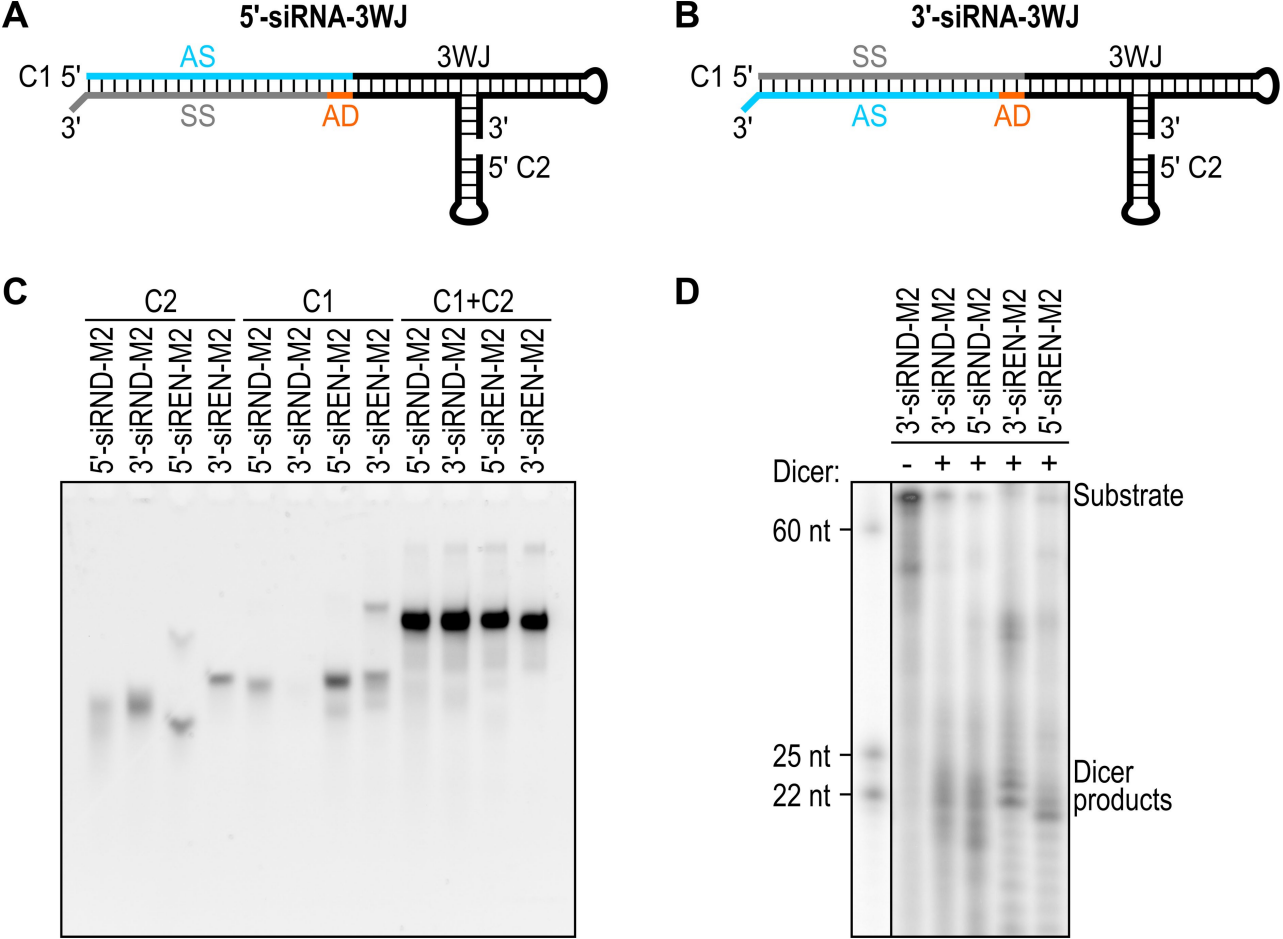
(A) Design for a 5′‐siRNA‐3WJ. (B) Design for a 3′‐siRNA‐3WJ. In (A and B), the antisense strand (AS) is blue, the sense strand (SS) is grey, an adapter (AD) is orange, and the 3WJ is black. Strands: C1, chimera 1; C2, chimera 2. (C) Non‐denaturing gel mobilities of Renilla luciferase‐targeting and control siRNA‐M2 constructs. The loading amount of each sample was matched at 1.67 pmol. RNA was stained with SYBR Gold Nucleic Acid Gel Stain, which detects double‐stranded nucleic acids more sensitively than single‐stranded nucleic acids.^[16]^ (D) Denaturing gel mobilities of Renilla luciferase‐targeting and control siRNA‐M2 constructs following incubation with (+) or without (−) recombinant human Dicer for 1 h at 37 °C. The 25‐ and 22‐nucleotide (nt) labels mark the expected range of Dicer cleavage products. The gel is representative of three independent replicates.

To determine whether the siRNA‐3WJs are substrates for Dicer, we 5′ end‐labeled C1, annealed C1 and C2, and incubated the siRNA‐3WJs with recombinant human Dicer for 1 h at 37 °C. siRNA‐3WJs incubated without Dicer served as negative controls. Following denaturing PAGE, small RNAs in the 22–25 nt range consistent with the products of Dicer cleavage^[26]^ were detected for all siRNA‐3WJs incubated with Dicer; no small RNAs were detected for siRNA‐3WJs incubated without Dicer (Figures 1D and S9). These results are consistent with studies showing that siRNAs fused to the full‐length phi29 pRNA or the phi29 3WJ are released by Dicer cleavage.[^10d^, ^15^]

To assess whether the siRNA‐3WJs require Dicer for gene silencing activity, we transfected Renilla luciferase‐targeting and control siRNA‐3WJs into wild‐type (WT) and Dicer‐deficient (NoDice) 293T cells, which were derived and characterized previously.^[23]^ Dicer deficiency was confirmed by western blot (Figure S10). After 24 h, we introduced a Renilla luciferase‐ and Firefly luciferase‐expressing plasmid. The activities of both Renilla and Firefly luciferase were measured 48 h post‐transfection with the plasmid. In WT cells, the Renilla luciferase‐targeting siRNA‐3WJs exhibited potent, dose‐dependent activity (Figure 2A). In NoDice cells, the siRNA‐3WJs retained potent, dose‐dependent activity (Figure 2B). In both WT and NoDice cells, the control siRNA‐3WJs were inactive (Figure 2C and D). The efficiencies of siRNAs fused to the phi29, M2, and SF5 3WJs were not significantly different at the concentrations tested (Figure S11). Interestingly, the positive control siREN and all Renilla luciferase‐targeting 5′‐siRNA‐3WJs were as efficient in NoDice cells as in WT cells (Figure S12). On the other hand, at 2.5 nM, all 3′‐siRNA‐3WJs were less efficient in NoDice cells than in WT cells (Figure S12).

**Figure 2:**
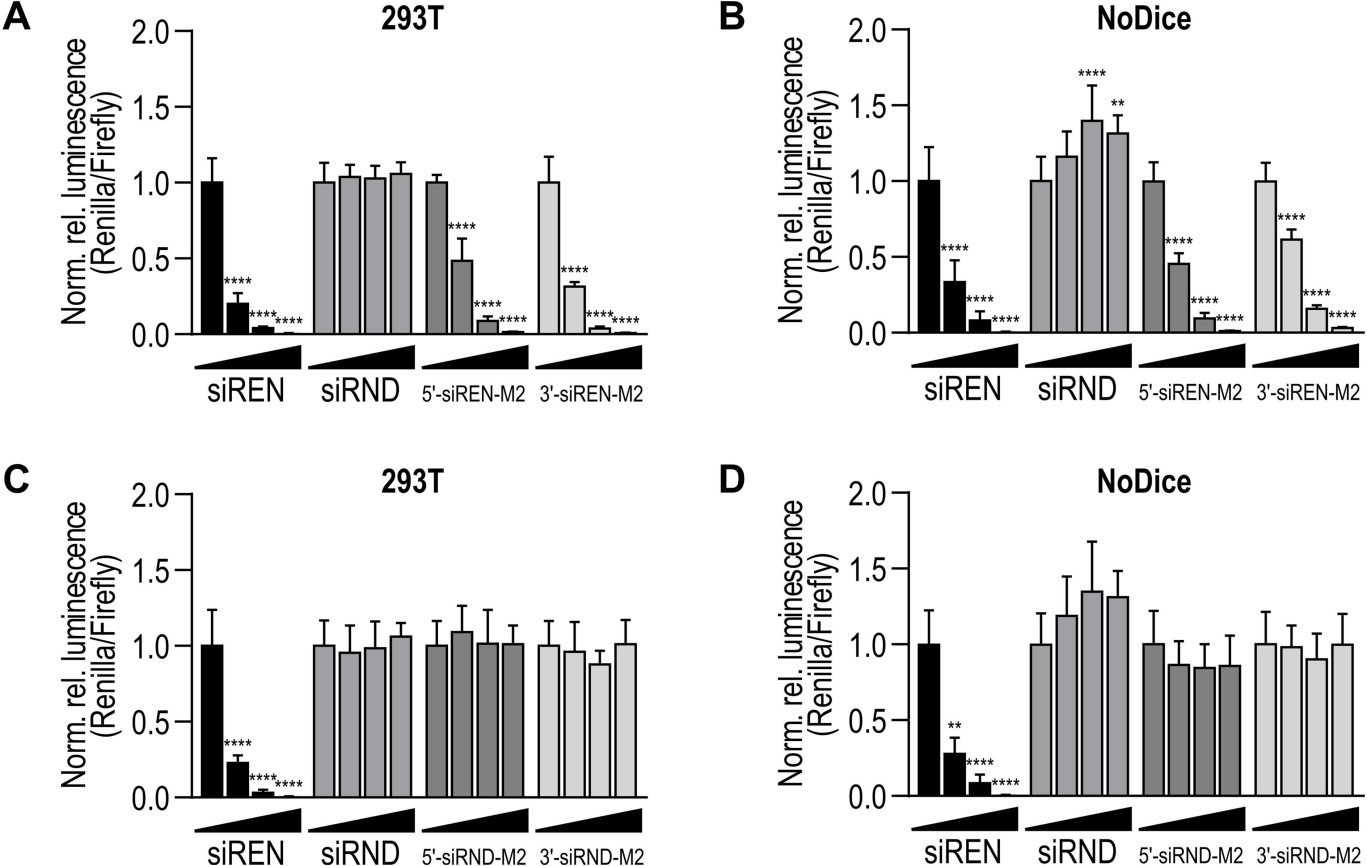
(A) Activity of Renilla luciferase‐targeting siRNA‐M2 constructs on a Renilla luciferase reporter in 293T cells. (B) Activity of Renilla luciferase‐targeting siRNA‐M2 constructs on the same reporter in Dicer‐deficient (NoDice) cells.^[23]^ (C) Activity of control siRNA‐M2 constructs on a Renilla luciferase reporter in 293T cells. (D) Activity of control siRNA‐M2 constructs on the same reporter in NoDice cells.^[23]^ In (A–D), siREN and siRND served as positive and negative controls. Treatments were transfected at 0, 2.5, 10, and 40 nM. Data are mean Renilla/Firefly values normalized to the 0 nM treatment ±SD (*n*=3). Statistics are two‐way ANOVA with Dunnett's multiple comparisons test against the 0 nM treatment: *P≤0.05, **P≤0.01, ***P≤0.001, ****P≤0.0001.

To investigate whether the location of the fused siRNA influences gene silencing efficiency, we designed additional siRNA‐3WJs using the M2 3WJ as a scaffold. The design described above (Figure 1A,B) was designated as design 1. Designs 2 and 3 were created by moving the siRNA clockwise around the 3WJ (Figure 3A). The new designs also use two strands (Table S5). RNAstructure software^[25]^ predicted that the paired strands fold as designed (Figure S13). Oligoribonucleotides were produced and assessed for self‐assembly using the aforementioned methods (Table S6, Figure S14). Dual‐luciferase reporter assays were performed as described above. In WT cells, the Renilla luciferase‐targeting siRNA‐3WJs exhibited potent, dose‐dependent activity (Figure 3B). In NoDice cells, they retained this activity (Figure 3C). The efficiencies of designs 1, 2, and 3 were not significantly different at the concentrations tested.

**Figure 3:**
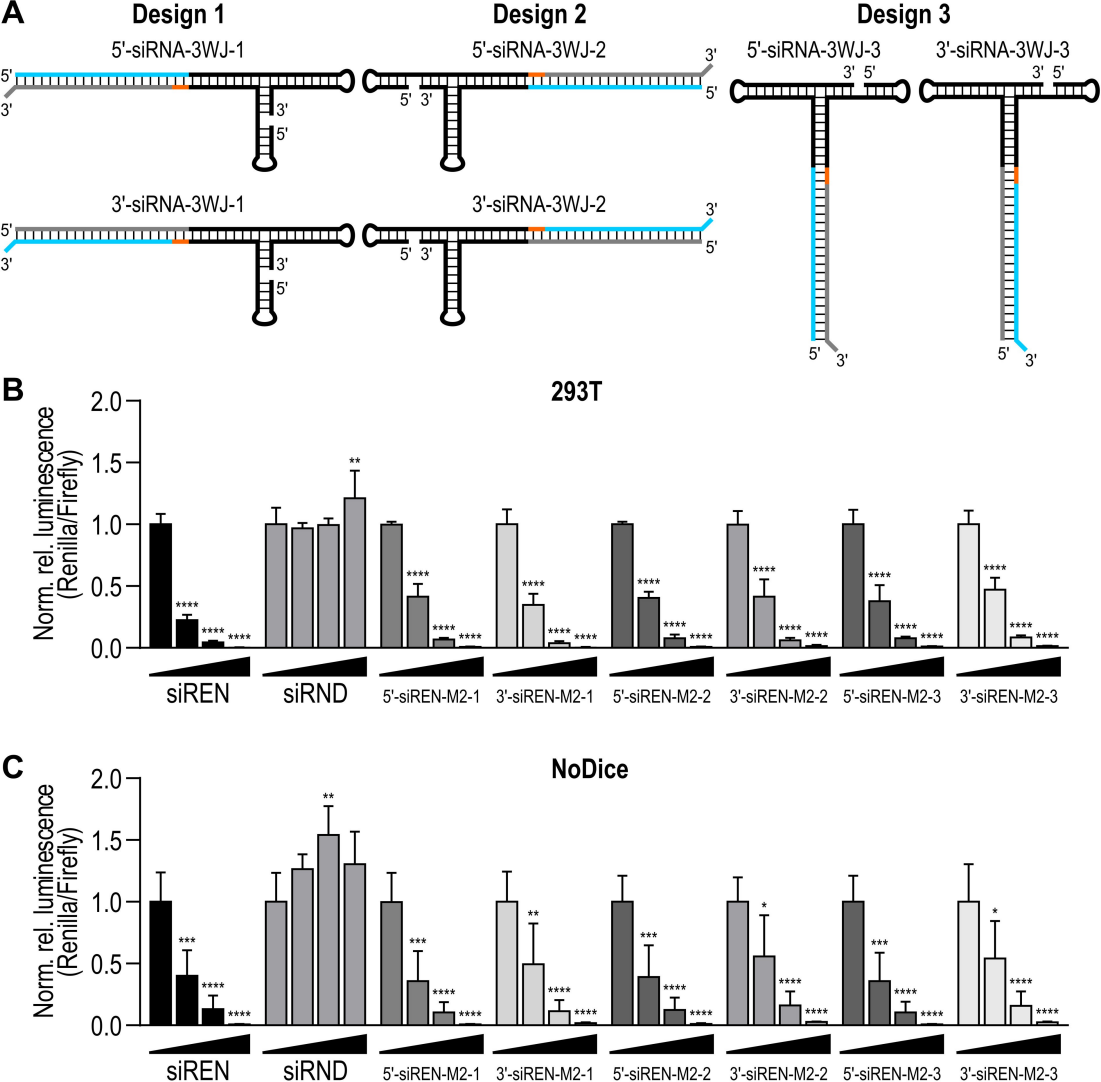
(A) siRNA‐3WJ designs 1 (left), 2 (middle), and 3 (right). The antisense strand (AS) is blue, the sense strand (SS) is grey, an adapter (AD) is orange, and the 3WJ is black. (B) Activity of Renilla luciferase‐targeting siRNA‐M2 construct designs 1, 2, and 3 on a Renilla luciferase reporter in 293T cells. (C) Activity of Renilla luciferase‐targeting siRNA‐M2 construct designs 1, 2, and 3 on the same reporter in Dicer deficient (NoDice) cells.^[23]^ In (B and C), siREN and siRND served as positive and negative controls. Treatments were transfected at 0, 2.5, 10, and 40 nM. Data are mean Renilla/Firefly values normalized to the 0 nM treatment ±SD (*n*=3). Statistics are two‐way ANOVA with Dunnett's multiple comparisons test against the 0 nM treatment: *P≤0.05, **P≤0.01, ***P≤0.001, ****P≤0.0001.

To confirm the Dicer‐independent gene silencing activity of the siRNA‐3WJs, we fused an siRNA targeting green fluorescent protein (GFP; siGFP) or a control siRNA (siPURO) to the M2 3WJ. These designs also use two strands (Table S7). RNAstructure software^[25]^ predicted that the paired strands fold as designed (Figure S15). Oligoribonucleotides were produced and assessed for self‐assembly using the aforementioned methods (Table S8, Figure S16). We transfected the siRNA‐3WJs into GFP‐expressing WT and Dicer‐knockout (KO) 293T cells. Dicer deficiency was confirmed by western blot (Figure S17). Notably, the level of Ago2 was strongly decreased in Dicer‐KO cells (Figure S17), which also has been shown previously.^[27]^ After 48 h, GFP expression was analyzed by flow cytometry. In WT cells, the GFP‐targeting siRNA‐3WJs exhibited potent, dose‐dependent activity (Figure 4A). In Dicer‐KO cells, both siRNA‐3WJs retained activity, although the 5′‐siRNA‐3WJ was more efficient than the 3′‐siRNA‐3WJ (Figure 4B). Transfection of the same siRNA‐3WJs into GFP‐expressing Ago2‐KO 293T cells (Figure S17) revealed that the siRNA‐3WJs retain some gene silencing activity in Ago2’s absence (Figure 4C). Again, the 5′‐siRNA‐3WJ was more efficient than the 3′‐siRNA‐3WJ (Figure 4C). Notably, siGFP was inactive in Ago2‐KO cells (Figure 4C).

**Figure 4:**
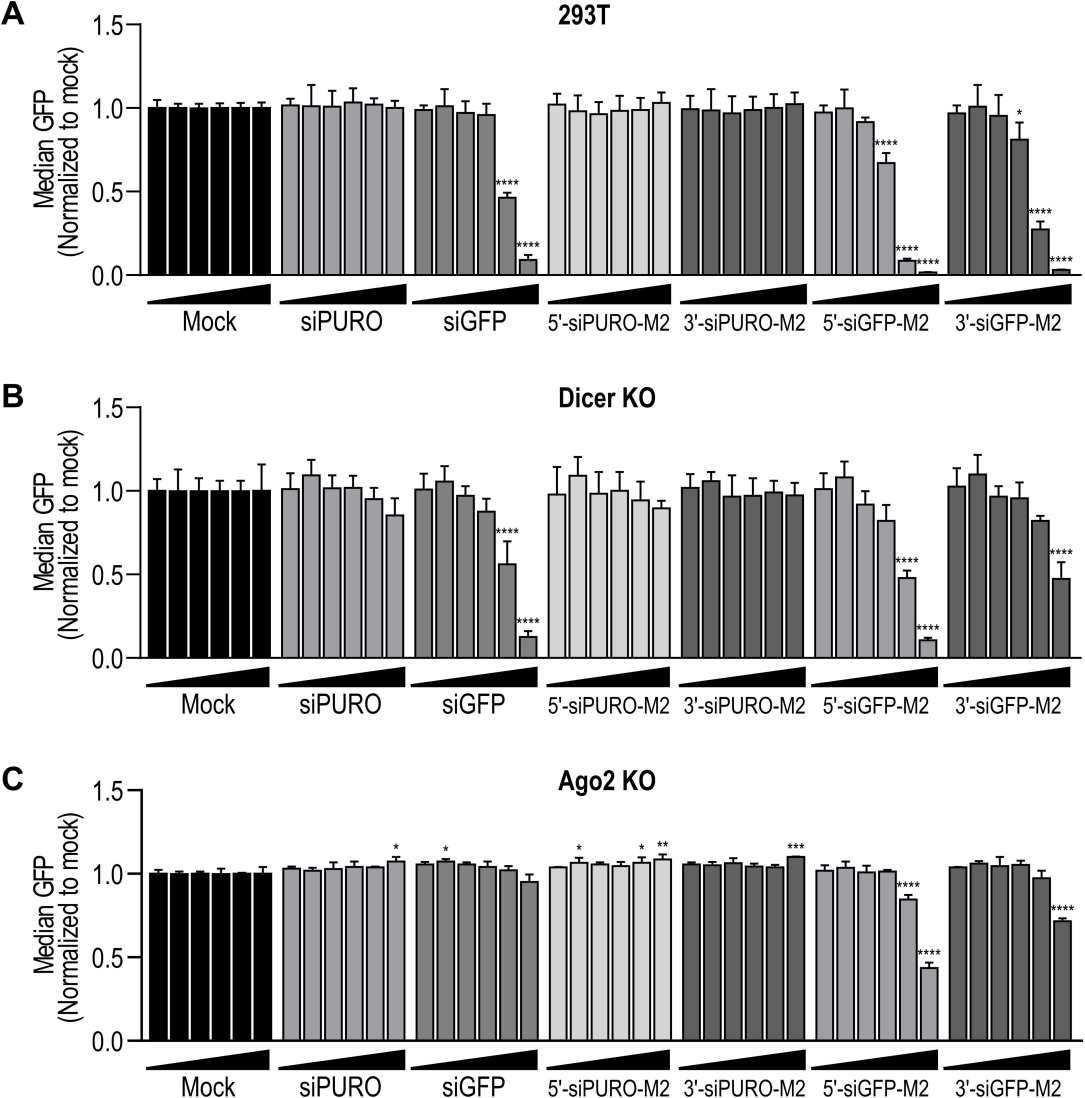
(A) Activity of GFP‐targeting and control siRNA‐M2 constructs in GFP‐expressing 293T cells. (B) Activity of GFP‐targeting and control siRNA‐M2 constructs in Dicer‐KO GFP‐expressing 293T cells. (C) Activity of GFP‐targeting and control siRNA‐M2 constructs in Ago2‐KO GFP‐expressing 293T cells. In (A–C), siGFP and siPURO served as positive and negative controls. Treatments were transfected at 0.01, 0.05, 0.1, 0.5, 2, and 10 nM. Mock was treated with the corresponding amount of transfection reagent alone. Data are mean GFP fluorescence normalized to the mock treatment ±SD (*n*=3). Statistics are two‐way ANOVA with Dunnett's multiple comparisons test against the mock treatment: *P≤0.05, **P≤0.01, ***P≤0.001, ****P≤0.0001.

Fusing siRNAs to the 3WJ derived from the phi29 bacteriophage pRNA is an innovative approach to siRNA delivery. In this study, we designed siRNA‐3WJs using the phi29 3WJ and two phylogenetically related 3WJs. Relative to the phi29 3WJ, the M2 and SF5 3WJs have higher thermodynamic stabilities.^[22]^ Our in vitro characterization of the 3WJs revealed that they have different thermal and serum stabilities (Table 1). The relationship between sequence and stability remains unclear, although the phi29 and M2 3WJs adopt distinct helical arrangements in solution^[28]^ that may contribute to differences in stability.^[29]^

We confirmed that siRNA‐3WJs are substrates for Dicer in vitro (Figures 1D and S9), consistent with studies showing that siRNAs fused to pRNA are released by Dicer cleavage.[^10d^, ^15^] However, siRNA‐3WJs transfected into Dicer‐deficient cell lines triggered potent gene silencing (Figures 2B, 3C and 4B). The activity of 5′‐siRNA‐3WJs in the absence of Dicer may be related to the Dicer‐independent activity observed for tripodal interfering RNA structures analogous to our 5′‐siRNA‐3WJ designs.^[10h]^ Alternatively, the activity of both siRNA‐3WJs in the absence of Dicer may be linked to the distinct Dicer‐independent mechanisms of gene silencing observed for short (≤19 bp) small hairpin RNAs (shRNAs).^[30]^ Specifically, short shRNAs with the antisense strand positioned 5′ of the terminal loop are loaded directly into RISC, where they are processed by Ago2.^[30]^ On the other hand, short shRNAs with the antisense strand positioned 3′ of the loop require terminal loop cleavage by another endogenous ribonuclease prior to RISC loading.^[30]^ Our 5′‐ and 3′‐siRNA‐3WJs may engage similar mechanisms, respectively, in Dicer's absence.

Interestingly, siRNA‐3WJs transfected into an Ago2‐deficient cell line retained some gene silencing activity (Figure 4C). We note that in our experimental setup, siRNA‐3WJs were generally more potent at silencing their target than their siRNA counterpart (Figure 4). This may partly explain why siRNA‐3WJs retain residual activity in Ago2‐KO cells, while siRNA activity is abrogated. We speculate that, in the absence of Ago2, siRNA‐3WJs associate with Ago1, Ago3, or Ago4. Only Ago2 has slicer activity and silences gene expression through catalytic cleavage; Ago1, Ago3, and Ago4 silence gene expression through translational repression and deadenylation.^[2]^ However, some guide RNAs confer slicer activity on Ago3 in vitro.^[31]^ Given that siRNA‐3WJs do not require Dicer for activity, and that they are active – albeit less potently – in the absence of Ago2, it is clear that their mechanism of gene silencing is more complicated than has been previously appreciated. It is possible that siRNA‐3WJs enter the RNAi pathway through multiple, interchangeable mechanisms, depending on the available machinery. Further studies will be required to explore this hypothesis.

The results presented here offer a design rule for maximizing the potency of siRNA‐3WJs in the human RNAi pathway. Our 5′‐siRNA‐3WJ was particularly active in Dicer‐deficient settings. Synthetic RNAi triggers that bypass Dicer cleavage have several advantages over those that depend on Dicer, including less protein kinase R/interferon induction, more precise processing and mRNA cleavage, and preferential association with Ago2.^[32]^ They also are more tolerant of chemical modifications^[33]^ that can improve pharmacokinetic and pharmacodynamic properties i*n vivo*. Thus, positioning the antisense strand of the siRNA duplex 5′ of the 3WJ may aid the development of pRNA 3WJs for siRNA delivery.

## Conflict of interest

A.C.H. is an inventor, applicant, and assignee listed on U.S. Patent No. 10,900,035 for pRNA 3WJ sequences.

## Supporting information

As a service to our authors and readers, this journal provides supporting information supplied by the authors. Such materials are peer reviewed and may be re‐organized for online delivery, but are not copy‐edited or typeset. Technical support issues arising from supporting information (other than missing files) should be addressed to the authors.

Supporting Information

## Data Availability

The data that support the findings of this study are available from the corresponding author upon reasonable request.
