# Supplementary material for: Chemically Synthesized, Self‐Assembling Small Interfering RNA‐Prohead RNA Molecules Trigger Dicer‐Independent Gene Silencing
Source: Chemistry. 2022 Jan 12;28(8):e202103995. doi: 10.1002/chem.202103995 (PMC9305526; doi:10.1002/chem.202103995)
Supplement: Supplementary file 1 — Supporting Information [file CHEM-28-0-s001.pdf]

Chemistry–A European Journal

Supporting Information

Chemically Synthesized, Self-Assembling Small Interfering RNA-Prohead RNA Molecules Trigger Dicer-Independent Gene Silencing

Alyssa C. Hill,* Daniël van Leeuwen, Verena Schlösser, Alok Behera, Bogdan Mateescu, and Jonathan Hall

Experimental methods

Design of three-way junction (3WJ) constructs

The phi29, M2, and SF5 3WJ constructs were designed as described previously.[1] Briefly, the constructs were designed to encompass the 3WJ region of folded prohead RNA (pRNA) using three component strands, which self-assemble *in vitro* when mixed in equimolar concentrations.[2] Strand 1 (S1), strand 2 (S2), and strand 3 (S3) retain as much sequence identity to wild-type (WT) pRNA sequences[3] as possible. Changes to WT sequences were made distal to the 3WJ "core" to ensure approximately equal free energies of helix formation among the three branches.

Design of small interfering RNA (siRNA)-3WJ constructs

A Renilla luciferase-targeting siRNA (siREN) or control siRNA (siRND)[4] was fused to the phi29, M2, and SF5 3WJ constructs on the S3/S1 helix. siRND has six randomized base-pair positions.[4] siREN also was fused to the S1/S2 and S2/S3 helices of the M2 3WJ. A green fluorescent protein (GFP)-targeting siRNA (siGFP) or control siRNA (siPURO) also was fused to the M2 3WJ on the S3/S1 helix. In each case, the antisense strand of the siRNA duplex was positioned 5' of the 3WJ to generate a 5'-siRNA-3WJ or 3' of the 3WJ to generate a 3'-siRNA-3WJ. A dinucleotide adapter was incorporated into the designs to account for a dinucleotide 3' overhang on the fused siRNA. The ends of the 3WJ helices not fused to an siRNA were joined by 5'-AAAA-3' tetraloops. A strand break was introduced approximately halfway into the full-length constructs to facilitate chemical synthesis, following a bipartite approach.[5] Thus, each siRNA-3WJ construct comprises two strands: chimera 1 (C1) and chimera 2 (C2). We used RNAstructure software[6] to predict the secondary structures of the designed siRNA-3WJ constructs.

Oligoribonucleotide synthesis and purification

The solid-phase synthesis of oligoribonucleotides was carried out on a MerMade 12 synthesizer (BioAutomation Corporation) in DMT-on mode using 2'-*O*-TBDMS phosphoramidites (Thermo Fisher Scientific) and Universal Unylinker Support controlled-pore glass solid support (ChemGenes). Oligoribonucleotides were cleaved from the solid support using gaseous methylamine at 70°C and 1.2 bar for 1.5 h and eluted with ethanol and water (1:1). Deprotection of the 2'-*O*-TBDMS group was carried out in dry 1-methyl-2-pyrrolidone, triethylamine, and triethylamine-trihydrofluoride (6:3:4) at 70°C for 2 h. The reaction was quenched with ethoxytrimethylsilane and diisopropylether. Following centrifugation, RNA pellets were resuspended in ultrapure water and purified by reverse-phase high performance liquid chromatography (RP-HPLC; Agilent 1200 Series, Agilent Technologies). Collected RNA was dried in a vacuum concentrator, and the DMT group was removed in 20% (v/v) acetic acid at room temperature for 15 min. The RNA was dried in vacuum concentrator and resuspended in ultrapure water prior to final, DMT-off RP-HPLC purification. Oligoribonucleotide mass and purity were verified by liquid chromatography-mass spectrometry (LC-MS) analysis on an Agilent 1200/6130 system equipped with a Waters Acquity OST C-18 column. Yield was assessed on a Nanodrop 2000 (Thermo Fisher Scientific). Oligoribonucleotides were stored in aqueous solution at -20°C.

Electrophoretic mobility shift assay (EMSA)

Component strands were annealed at 2 μM in 1 M sodium chloride, 10 mM sodium cacodylate, 0.5 mM ethylenediaminetetraacetic acid (EDTA), pH 7.0 by heating to 95°C for 1 min followed by cooling at a rate of 1°C/min to a final temperature of 4°C on a C1000 Touch Thermal Cycler (Bio-Rad). Annealed samples were diluted 1:10 in ultrapure water and mixed with Gel Loading Dye, Purple (6X), no SDS (New England BioLabs) prior to 12% or 20% non-denaturing polyacrylamide gel electrophoresis (PAGE) at 4°C. The loading amount of each sample was matched at 1.67 pmol. RNA was stained with SYBR Gold Nucleic Acid Gel Stain (Thermo Fisher Scientific) and visualized by UV transillumination on a Gel Doc XR+ Gel Documentation System (Bio-Rad).

UV melting

Component strands were mixed at 2 µM in 1 M sodium chloride, 10 mM sodium cacodylate, 0.5 mM EDTA, pH 7.0. Melting curves were obtained by measuring absorbance at 260 nm over the temperature range 5°C to 90°C on a CARY 300 spectrophotometer (Varian, Inc.). The heating rate was 0.5°C/min and the data collection interval was 0.5°C. Each temperature series was performed three times with hold periods of 5 min at both 5°C and 90°C to ensure thermal equilibrium. Mean melting temperatures (T_ms) were determined from first derivative analyses of nonlinear fit melting curves in Microsoft Excel.

Serum stability assay

Component strands were annealed at 50 µM in 1 M sodium chloride, 10 mM sodium cacodylate, 0.5 mM EDTA, pH 7.0 by heating to 95°C for 1 min followed by cooling at a rate of 1°C/min to a final temperature of 4°C on a C1000 Touch Thermal Cycler (Bio-Rad). Annealed samples were diluted 1:1 in mouse serum (Sigma-Aldrich) to a final concentration of 25 µM RNA and 50% serum and then incubated at 37°C. At 0, 1, 2, 4, 6, 8, and 24 h, 30 µL samples were mixed with 66 µL MasterPure Tissue & Cell Lysis Solution (Epicentre) and digested with 4 µL Proteinase K (20 mg/mL; Roche) at 65°C for 30 min. As controls, a mock sample (25 µM RNA in ultrapure water) and a blank sample (50% serum) were incubated at 37°C for 24 h and processed as described above. Quenched mixtures were stored at -80°C. Prior to analysis, samples were thawed on ice and denatured at 95°C for 5 min. SDS was precipitated with 20 µL 3 M potassium chloride and pelleted by centrifugation. Supernatants were diluted 1:10 in ultrapure water, mixed with Gel Loading Dye, Purple (6X), no SDS (New England BioLabs), and subjected to 20% non-denaturing PAGE at 4°C. RNA was stained with SYBR Gold Nucleic Acid Gel Stain (Thermo Fisher Scientific) and visualized by UV transillumination on a Gel Doc XR+ Gel Documentation System (Bio-Rad). For analysis, the intensity of the 3WJ band at each time point was measured using ImageJ software.[7] Background-corrected intensity values were normalized to the 0 h time point and averaged for three independent replicates. Half-lives ($t_{½}$) were calculated from normalized intensity data fit to one-phase exponential decay equations with plateau = 0 in GraphPad Prism version 8.2.0 for Windows (GraphPad Software, San Diego, California USA).

In vitro Dicer cleavage assay

In vitro Dicer cleavage assays were performed as described previously.[8] Briefly, C1 was prepared at 1 µM in ultrapure water, 5' end-labeled with [γ-^{32}P] ATP (Perkin Elmer, Cat. No. BLU002Z250UC) and T4 polynucleotide kinase (PNK; New England BioLabs, Cat. No. M0201S), and denatured at 95°C for 5 min. C2 was prepared at 0.1 µM in ultrapure water and dried in a vacuum concentrator. C2 samples were resuspended in C1 labeling reactions and annealed by heating to 95°C for 1 min in an Eppendorf Thermomixer Compact thermoblock (Sigma-Aldrich) and cooling to ambient temperature. Single-turnover Dicer cleavage assays were performed with 20 fmol [^{32}P]-labeled siRNA-3WJ constructs and 300 fmol recombinant Dicer (OriGene, NM_177438, Cat. No. TP319214) in 25 mM Tris, pH 7, 25 mM NaCl, 1 mM DTT, 2 mM $MgCl_2$, and 1% glycerol. Samples were incubated at 37°C for 1 h and separated by 20% denaturing urea-PAGE (SequaGel, National Diagnostics). Single-stranded RNA ladder: 60-nt oligomer (5'-AAGGAUGACACGCAAAUUCGUGACGUACGUACGUACGUACGUACGUACGUACGUAC-3'), 25-nt oligomer (5'-AAGGAUGACACGCAAAUUCGUGACG-3'), 22-nt oligomer (5'-AAGGAUGACACGCAAAUUCGUG-3'). Gels were placed on a phosphor screen overnight, and signals were measured on a Typhoon FLA 7000 phosphor imager (GE Healthcare Life Sciences).

Western blotting

Protein extracts (30 µg) from WT 293T, NoDice(2-20), and NoDice(4-25) cells[9] were separated by 8% SDS-PAGE and transferred to a nitrocellulose blotting membrane with a pore size of 0.45 µm (GE Healthcare, Cat. No. 10600008) using a Mini Trans-Blot® Cell wet transfer device (Bio-Rad). The proteins were detected using an anti-Dicer antibody (Santa Cruz Biotechnology, Cat.

No. sc-136979; 1:1000 dilution) and anti-GAPDH antibody (Proteintech, Cat. No. 60004-1-Ig; 1:5000 dilution). Western blots were revealed using the ECL™ Prime Western Blotting Detection Reagent (Amersham, Cat. No. RPN2232) on a ChemiDoc XRS+ System (Bio-Rad).
Proteins extracted from approximately 3 x 10^6 GFP-expressing cells in RIPA buffer were diluted to 15 µg together with 4X western blot loading dye (250 mM Tris-HCl, pH 6.8, 40% glycerol, 20% 2-β-mercaptoethanol, 16% SDS) and heated at 95°C for 5 min before loading with 5 µL Pageruler Plus Prestained Protein Ladder (Thermo Scientific) on the corresponding % acrylamide gel: 12% for GAPDH, 10% for Argonaute 2 (Ago2), and 6% for Dicer detection. Samples were run in Mini-Trans Blot cells (Bio-Rad) at 80 V through the stacking gel and 120 V in the resolving gel. Transfers were performed at 100 V and 4°C for 120 min. Proteins were detected using anti-Argonaute 2 (Cell Signaling Technology, Cat. No. 2897; 1:1000 dilution), anti-Dicer (Sigma-Aldrich, Cat. No. SAB4200087; 1:5000 dilution), and anti-Tubulin (Sigma-Aldrich, Cat. No. T6199; 1:10000 dilution) antibodies. Blots were revealed with the Lumi-light Plus Western Blot Substrate (Roche) on a ChemiDoc (Bio-Rad).

Cell culture

WT 293T and NoDice(4-25) cells[9] were maintained at 37°C with 5% CO_2 in Dulbecco's Modified Eagle's Medium (DMEM; Thermo Fisher Scientific) supplemented with 10% fetal bovine serum (FBS; Gibco). WT (pOSI17 WT, kind gift of Prof. Michael McManus, UCSF), Dicer-knockout (KO; pOSI17 $Dicer^{-/-}$), and Ago2-KO (pOSI17 $Ago2^{-/-}$) GFP-expressing 293T cells were maintained at 37°C with 5% CO_2 in DMEM (Thermo Fisher Scientific) supplemented with 10% FBS and penicillin-streptomycin solution (Sigma-Aldrich).

Dual-reporter luciferase assay

Cells were seeded in DMEM + 10% FBS at 10,000 live cells/well in opaque, 96-well plates (Thermo Fisher Scientific). Transfections were performed 8 h post-seeding using Lipofectamine 2000 Transfection Reagent (Invitrogen) according to the manufacturer's instructions. Transfections with 20 ng reporter plasmid/well were performed 24 h later using jet-PEI (Polyplus-transfection) according to the manufacturer's instructions. Luciferase assays were performed 48 h post-plasmid transfection using the Dual-Glo® Luciferase Assay System (Promega). Luminescence counts were measured on a Mithras LB 940 plate reader (Berthold Technologies). Relative luminescence values (Renilla/Firefly) were normalized to the 0 nM treatment and averaged for three biological replicates. Statistical analyses were performed in GraphPad Prism version 8.2.0 for Windows (GraphPad Software, San Diego, California USA).

Generation of Dicer-KO and Ago2-KO GFP-expressing 293T cells

gRNAs were cloned into the lentiCRISPRv2 backbone as described previously.[10] LentiCRISPRv2 was a gift from Feng Zhang. Correct gRNA insertion into LentiCRISPRv2 was validated by Sanger sequencing (Eurofins Genomics) with the hU6-F primer. Oligos used for cloning the gRNAs into the lentiCRISPRv2 backbone are described in Table S9.
LentiCRISPRv2 transfections in HEK293T cells were performed with Viafect (Promega) to generate Ago2 and Dicer CRISPR-Cas9 knockouts. One day before transfection, 4 x 10^5 HEK293T pOSI17 cells were seeded in a 6-well plate. Two days after transfection, cells were split to a 10-cm dish. Five days after transfection, cells were counted and seeded at 0.5 cells/well in a 96-well plate to grow single clones. After approximately two weeks in the 96-well plate, clones were picked and expanded. During clone expansion, the gDNA was checked for Cas9 activity with a T7E1 assay. In short, gDNA was extracted with the KAPA Express Extract kit (Sigma-Aldrich), and the sequence around the guide was PCR amplified, purified, and cut with T7E1 enzyme (Thermo Scientific). Later, when cells were expanded, the T7E1 results were validated by western blot. Oligos used for PCR amplification of the gDNA are described in Table S10.

Flow cytometry analysis

WT, Dicer-KO, and Ago2-KO GFP-expressing 293T cells were seeded at 10,000 cells/well in 96-well plates in 200 µL of medium. At 24 h post-seeding, 10 µL of corresponding transfection mixture was added to the cells in 100 µL of medium. Cells were transfected using Lipofectamine 3000 Reagent (Thermo Fisher Scientific) according to the manufacturer's instructions. In short, per condition per well, 0.35 µl of Lipofectamine 3000 was mixed with 10 nM of RNA in a total volume of 10 µl basic medium. Serial dilutions were made from the 10 nM mixture to obtain the 0.01, 0.05, 0.1, 0.5, and 2 nM concentrations. At 24 h post-transfection, 100 µL of medium was added to the wells. After 48 hours, cells were detached and dissociated with trypsin, resuspended in 100 µL of cell culture medium, and analyzed by flow cytometry on a CytoFLEX S Flow Cytometer (Beckman Coulter). FSC-A and SSC-A parameters were used to separate the cell population from debris, and then FSC-H and FSC-A were used to select 'singlets' cell population. We acquired the median GFP signal (laser: 488 nm, emission filter: 525/40 nm) of >1 x 10^4 singlet events in order to quantify GFP silencing efficiency for each condition. Experiments were performed in biological triplicate and the mean of three median GFP values is displayed in the graphs with corresponding standard deviations. Statistical analyses were performed in GraphPad Prism version 8.2.0 for Windows (GraphPad Software, San Diego, California USA).

Table S1. Sequences of 3WJ constructs. Changes to wild-type pRNA sequences[3] are bold.

Construct	Strand	Sequence (5' to 3')
Phi29 3WJ	1	**C**UUGUCAUGUGUAUGUUG**CC**
	2	**GG**CACAUACUUUGUUGAU**A**GG
	3	**CC**UGUCAAUCAUGGCAA**G**
M2 3WJ	1	**G**C**A**AUAGUAUGGCACAUG**UGC**
	2	**G**CACAUGUCACGGGG**UAC**G
	3	**CGU**ACCCUCUUACUA**UUGC**
SF5 3WJ	1	**GC**UAAUGUAUGUGUGUC**C**G
	2	C**G**GACAGCAGGGGAG**GUGC**
	3	**GCAC**CUCUUGCAUUA**GC**

Table S2. Calculated and measured masses of 3WJ construct component strands.

Construct	Strand	Calculated mass (g/mol)	Measured mass (g/mol)
Phi29 3WJ	1	6298.8	6298.02
	2	6714.1	6713.23
	3	5715.5	5714.76
M2 3WJ	1	6736.1	6735.44
	2	6115.8	6115.01
	3	5895.5	5894.83
SF5 3WJ	1	6055.7	6054.91
	2	6233.9	6233.08
	3	5323.2	5322.51

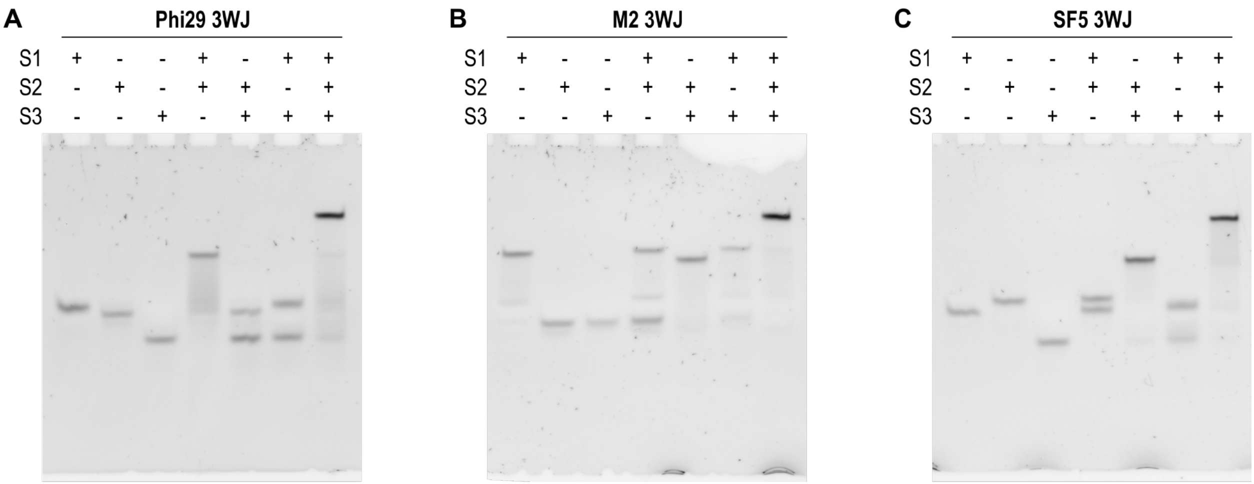

Figure S1. (A) 20% non-denaturing polyacrylamide gel mobility of the phi29 3WJ. (B) 20% non-denaturing polyacrylamide gel mobility of the M2 3WJ. (C) 20% non-denaturing polyacrylamide gel mobility of the SF5 3WJ. The loading amount of each sample was matched at 1.67 pmol. RNA was stained with SYBR Gold Nucleic Acid Gel Stain, which detects double-stranded nucleic acids more sensitively than single-stranded nucleic acids.[11]

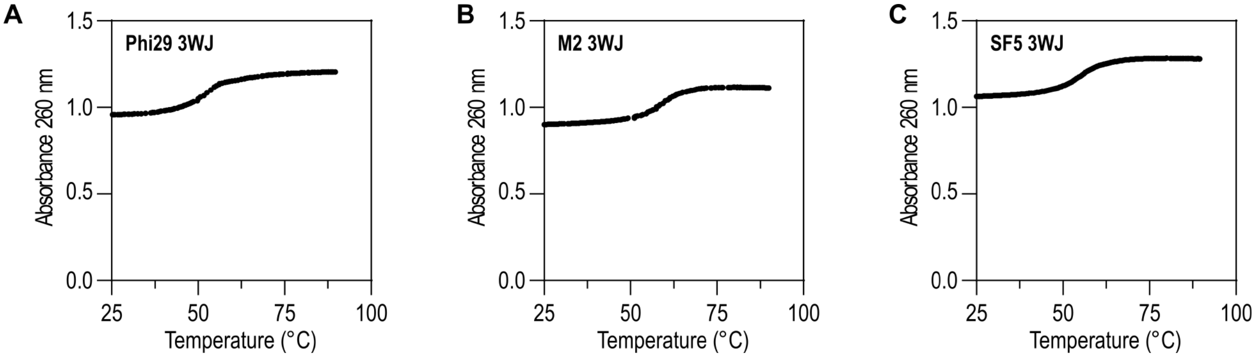

Figure S2. (A) Phi29 3WJ melting curve. (B) M2 3WJ melting curve. (C) SF5 3WJ melting curve. Data are representative of three temperature series.

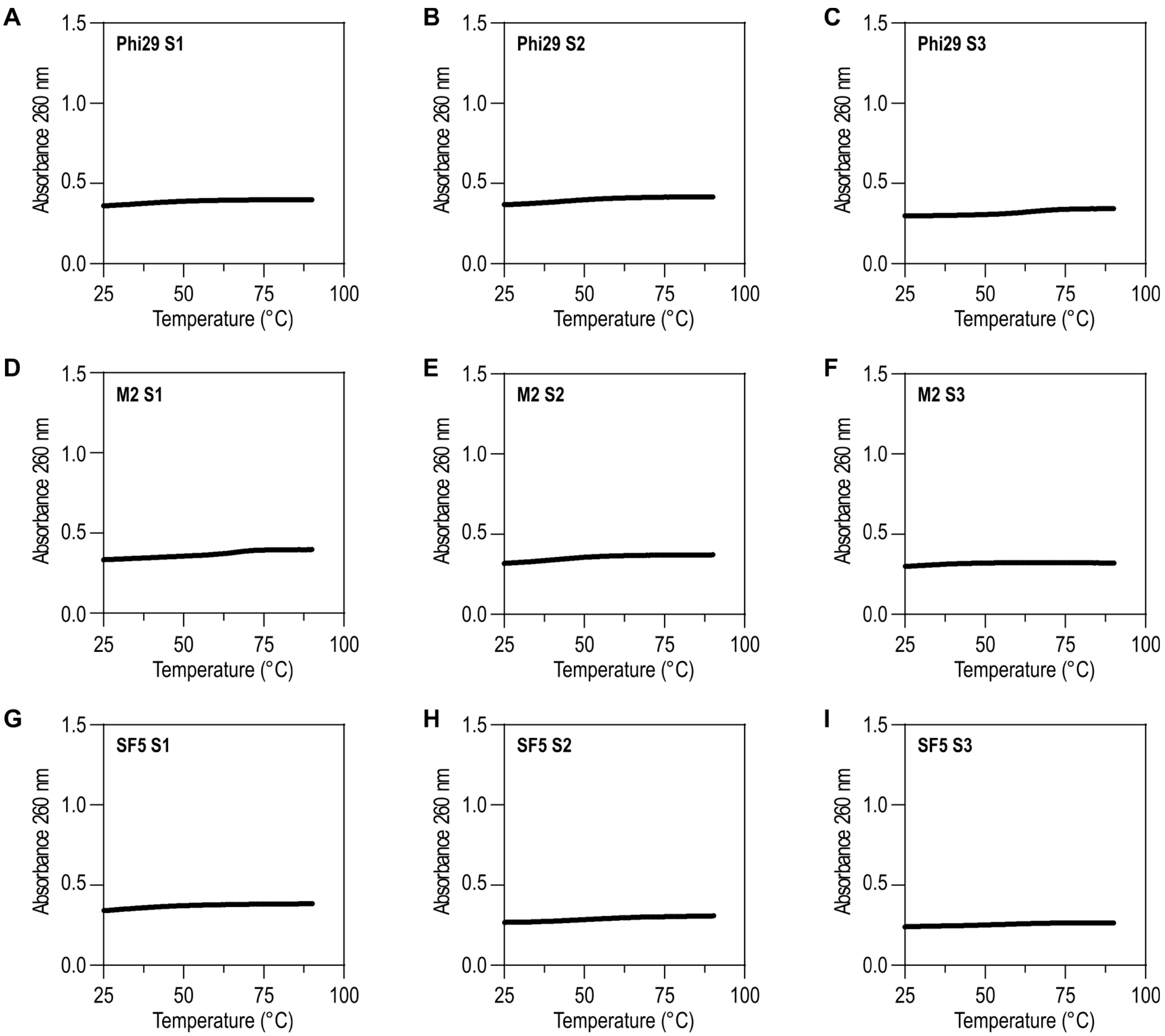

Figure S3. (A) Phi29 S1 melting curve. (B) Phi29 S2 melting curve. (C) Phi29 S3 melting curve. (D) M2 S1 melting curve. (E) M2 S2 melting curve. (F) M2 S3 melting curve. (G) SF5 S1 melting curve. (H) SF5 S2 melting curve. (I) SF5 S3 melting curve. Data are representative of three temperature series.

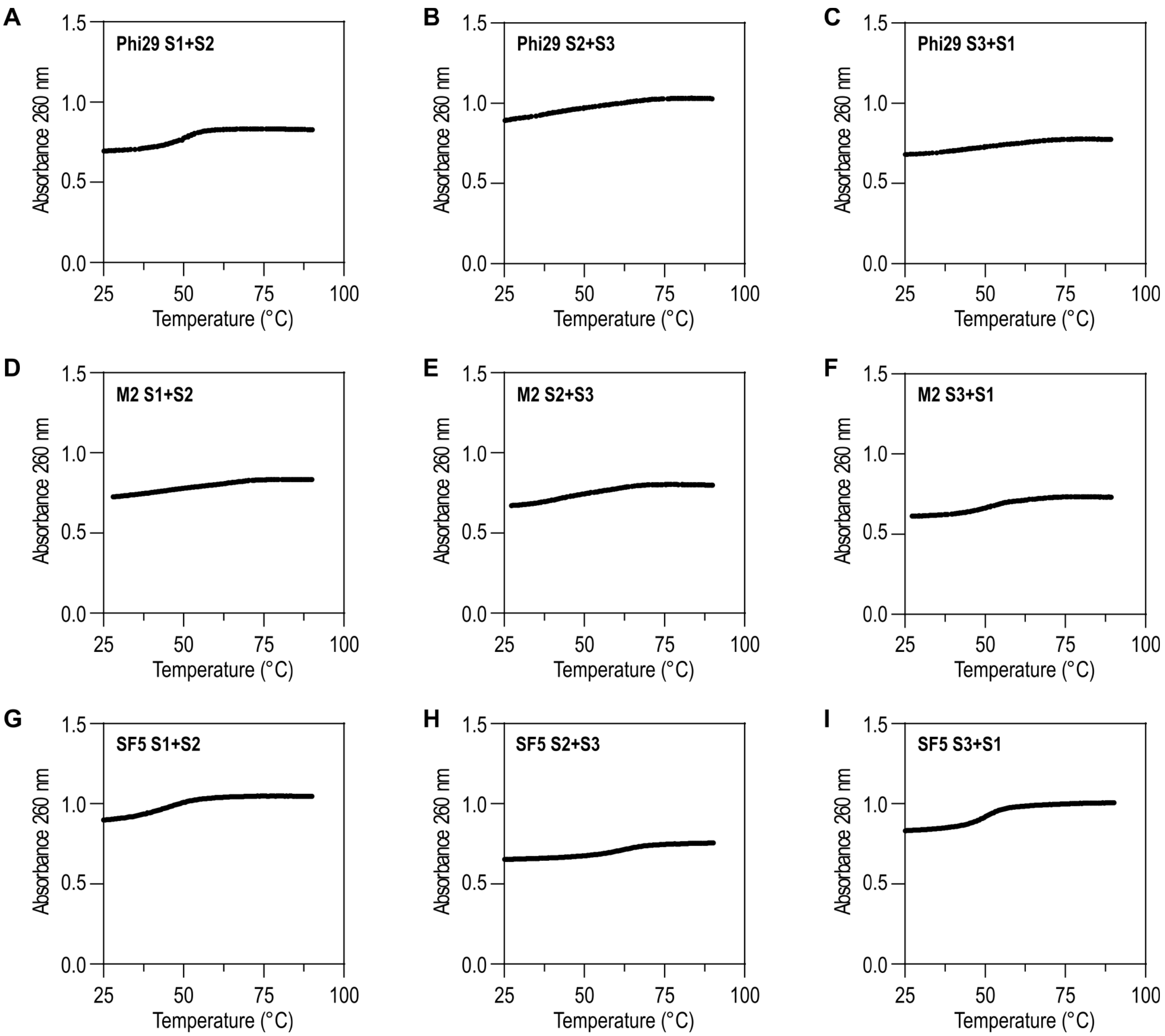

Figure S4. (A) Phi29 S1+S2 melting curve. (B) Phi29 S2+S3 melting curve. (C) Phi29 S3+S1 melting curve. (D) M2 S1+S2 melting curve. (E) M2 S2+S3 melting curve. (F) M2 S3+S1 melting curve. (G) SF5 S1+S2 melting curve. (H) SF5 S2+S3 melting curve. (I) SF5 S3+S1 melting curve. Data are representative of three temperature series.

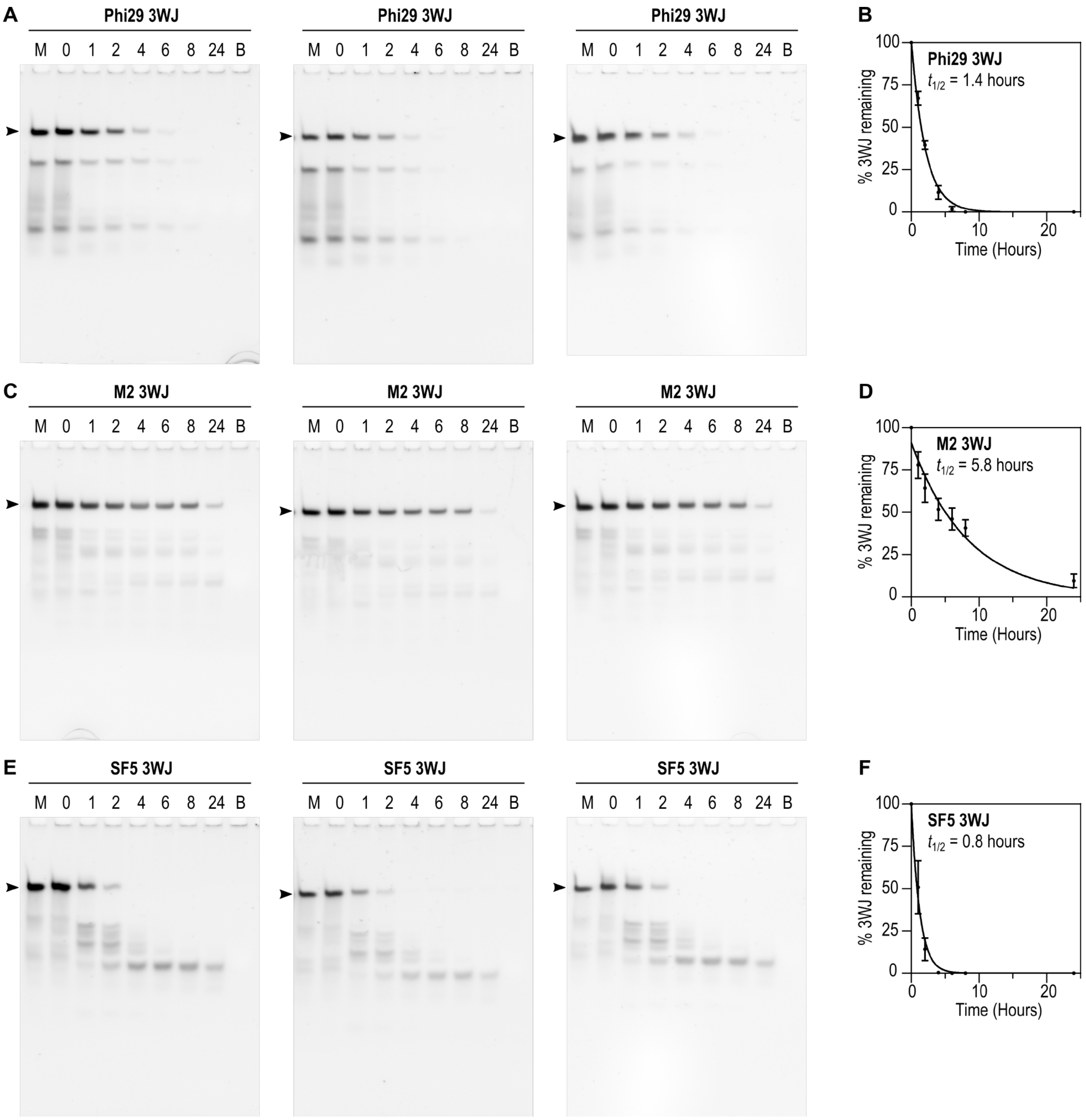

Figure S5. (A) Serum stability assays for the phi29 3WJ. (B) Phi29 3WJ exponential decay curve. (C) Serum stability assays for the M2 3WJ. (D) M2 3WJ exponential decay curve. (E) Serum stability assays for the SF5 3WJ. (F) SF5 3WJ exponential decay curve. Gel images depict 20% non-denaturing polyacrylamide gel mobilities following incubation in 50% mouse serum. Hours of incubation are indicated above each lane. M, mock; B, blank. Arrowheads indicate the bands used for quantification. Data in the graphs are mean percentage 3WJ remaining at each time point relative to the 0 h time point ± SD (n=3). $t_{½}$, half-life.

Table S3. Sequences of Renilla luciferase-targeting and control siRNA-3WJ constructs. Antisense sequences are underlined. Adapters are bold. X, random nucleotide.

Construct	Fusion helix	Strand	Sequence (5' to 3')
5'-siREN-phi29	S3/S1	C1	UUUCUCGCCCUCUUCGCUCUUCUUGUCAUGUGUAUGUUGCCAAAAGGCACAUACUUUGUUG
	S3/S1	C2	AUAGGAAAACCUGUCAAUCAUGGCAAG**AA**GAGCGAAGAGGGCGAGAAAUU
3'-siREN-phi29	S3/S1	C1	GAGCGAAGAGGGCGAGAAAUUCUUGUCAUGUGUAUGUUGCCAAAAGGCACAUACUUUGUUG
	S3/S1	C2	AUAGGAAAACCUGUCAAUCAUGGCAAG**AA**UUUCUCGCCCUCUUCGCUCUU
5'-siRND-phi29	S3/S1	C1	GUXUGXGUUXXUUAXXCACUUCUUGUCAUGUGUAUGUUGCCAAAAGGCACAUACUUUGUUG
	S3/S1	C2	AUAGGAAAACCUGUCAAUCAUGGCAAG**AA**GUGXXUAAXXAACXCAXACUU
3'-siRND-phi29	S3/S1	C1	GUGXXUAAXXAACXCAXACUUCUUGUCAUGUGUAUGUUGCCAAAAGGCACAUACUUUGUUG
	S3/S1	C2	AUAGGAAAACCUGUCAAUCAUGGCAAG**AA**GUXUGXGUUXXUUAXXCACUU
5'-siREN-M2	S3/S1	C1	UUUCUCGCCCUCUUCGCUCUUGCAAUAGUAUGGCACAUGUGCAAAAGCACAUGUCACGGGG
	S3/S1	C2	UAGGAAAACCUACCCUCUUACUAUUGC**AA**GAGCGAAGAGGGCGAGAAAUU
3'-siREN-M2	S3/S1	C1	GAGCGAAGAGGGCGAGAAAUUGCAAUAGUAUGGCACAUGUGCAAAAGCACAUGUCACGGGG
	S3/S1	C2	UAGGAAAACCUACCCUCUUACUAUUGC**AA**UUUCUCGCCCUCUUCGCUCUU
5'-siRND-M2	S3/S1	C1	GUXUGXGUUXXUUAXXCACUUGCAAUAGUAUGGCACAUGUGCAAAAGCACAUGUCACGGGG
	S3/S1	C2	UAGGAAAACCUACCCUCUUACUAUUGC**AA**GUGXXUAAXXAACXCAXACUU
3'-siRND-M2	S3/S1	C1	GUGXXUAAXXAACXCAXACUUGCAAUAGUAUGGCACAUGUGCAAAAGCACAUGUCACGGGG
	S3/S1	C2	UAGGAAAACCUACCCUCUUACUAUUGC**AA**GUXUGXGUUXXUUAXXCACUU
5'-siREN-SF5	S3/S1	C1	UUUCUCGCCCUCUUCGCUCUUGCUAAUGUAUGUGUGUCCGAAAACGGACAGCAGGGGAGC
	S3/S1	C2	GUCGAAAACGACACUCUUGCAUUAGC**AA**GAGCGAAGAGGGCGAGAAAUU
3'-siREN-SF5	S3/S1	C1	GAGCGAAGAGGGCGAGAAAUUGCUAAUGUAUGUGUGUCCGAAAACGGACAGCAGGGGAGC
	S3/S1	C2	GUCGAAAACGACACUCUUGCAUUAGC**AA**UUUCUCGCCCUCUUCGCUCUU
5'-siRND-SF5	S3/S1	C1	GUXUGXGUUXXUUAXXCACUUGCUAAUGUAUGUGUGUCCGAAAACGGACAGCAGGGGAGC
	S3/S1	C2	GUCGAAAACGACACUCUUGCAUUAGC**AA**GUGXXUAAXXAACXCAXACUU
3'-siRND-SF5	S3/S1	C1	GUGXXUAAXXAACXCAXACUUGCUAAUGUAUGUGUGUCCGAAAACGGACAGCAGGGGAGC
	S3/S1	C2	GUCGAAAACGACACUCUUGCAUUAGC**AA**GUXUGXGUUXXUUAXXCACUU

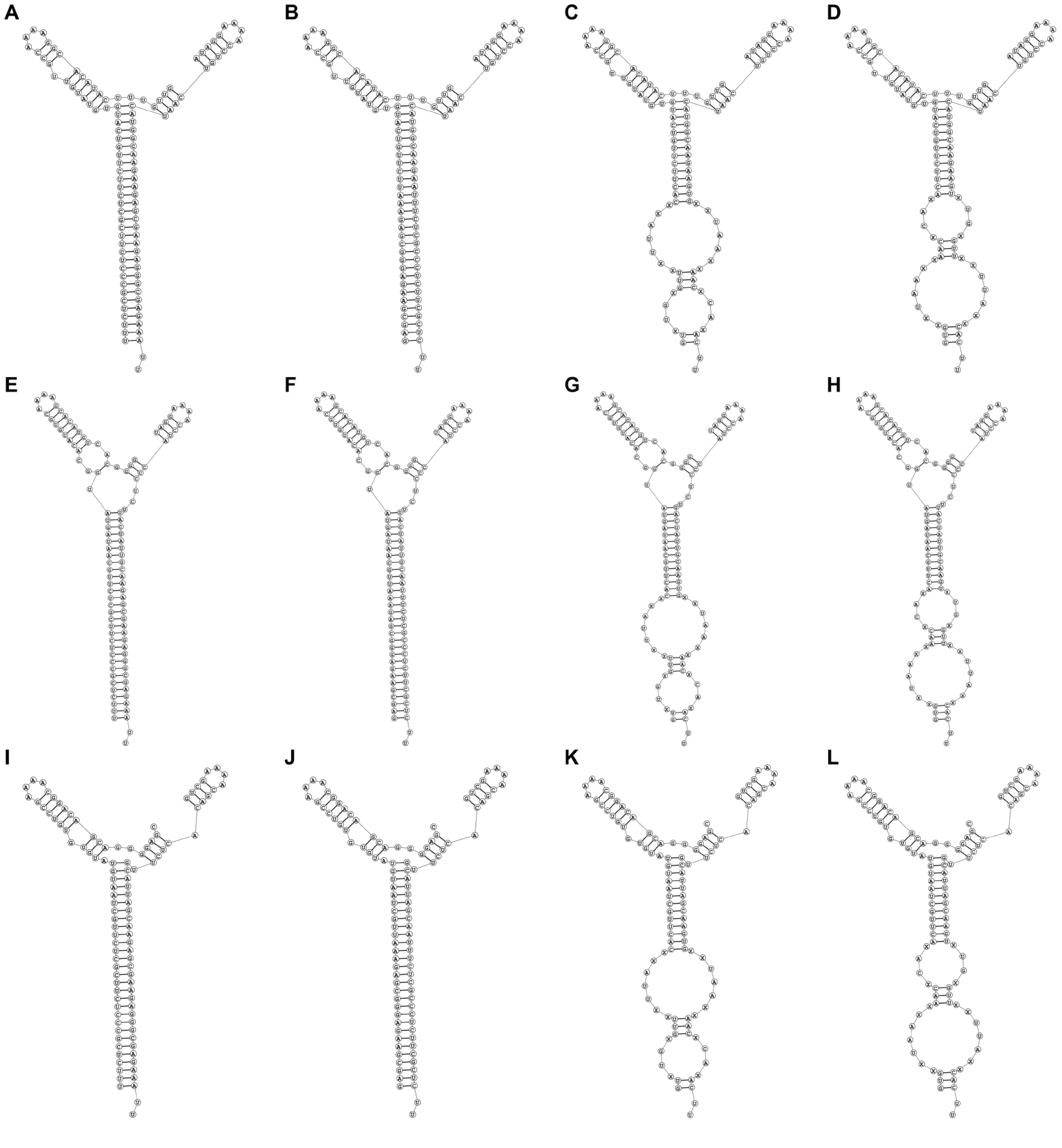

Figure S6. (A) Minimum free energy prediction for 5'-siREN-phi29. (B) Minimum free energy prediction for 3'-siREN-phi29. (C) Minimum free energy prediction for 5'-siRND-phi29. (D) Minimum free energy prediction for 3'-siRND-phi29. (E) Minimum free energy prediction for 5'-siREN-M2. (F) Minimum free energy prediction for 3'-siREN-M2. (G) Minimum free energy prediction for 5'-siRND-M2. (H) Minimum free energy prediction for 3'-siRND-M2. (I) Minimum free energy prediction for 5'-siREN-SF5. (J) Minimum free energy prediction for 3'-siREN-SF5. (K) Minimum free energy prediction for 5'-siRND-SF5. (L) Minimum free energy prediction for 3'-siRND-SF5. Predictions were made using the RNAstructure web servers version 6.0.1.[6] Results are bifold calculations on RNA using default parameters. siRND has six randomized base-pair positions.[4] The single-stranded randomized RNAs hybridize to form the most thermodynamically stable (*i.e.*, complementary) duplexes[4] and were not expected to interfere with the formation of the siRNA-3WJ structures. Nucleotides at randomized positions were input as unknown nucleotides (X) and appear unpaired in the figures.

Table S4. Calculated and measured masses of Renilla luciferase-targeting and control siRNA-3WJ construct component strands.

Construct	Fusion helix	Strand	Calculated mass (g/mol)	Measured mass (g/mol)
5’-siREN-phi29	S3/S1	C1	19235.4	19238.48
	S3/S1	C2	16309.0	16307.35
3’-siREN-phi29	S3/S1	C1	19699.9	19699.10
	S3/S1	C2	15844.5	15843.58
5’-siREN-M2	S3/S1	C1	19443.7	19445.20
	S3/S1	C2	16119.8	16118.45
3’-siREN-M2	S3/S1	C1	19908.2	19907.00
	S3/S1	C2	15655.3	15657.27
5’-siREN-SF5	S3/S1	C1	19170.5	19169.66
	S3/S1	C2	15892.7	15891.40
3’-siREN-SF5	S3/S1	C1	19635.0	19634.31
	S3/S1	C2	15428.2	15427.18

Oligoribonucleotides synthesized with random nucleotide positions contain up to 4^6 = 4,096 unique sequences with up to 84 unique masses.[4] For these oligoribonucleotides, the measured masses fall within the expected range, *i.e.*, between the mass calculated for a sequence with G nucleotides at all randomized positions and the mass calculated for a sequence with C nucleotides at all randomized positions.

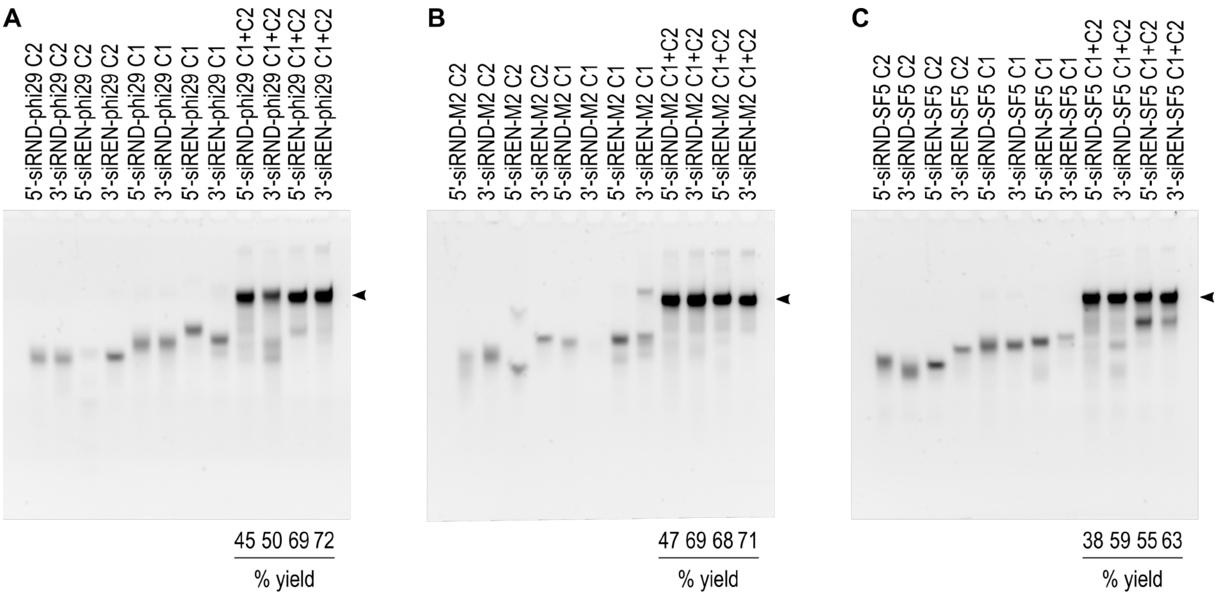

Figure S7. (A) 12% non-denaturing polyacrylamide gel mobilities of Renilla luciferase-targeting and control siRNA-phi29 constructs. (B) 12% non-denaturing polyacrylamide gel mobilities of Renilla luciferase-targeting and control siRNA-M2 constructs. (C) 12% non-denaturing polyacrylamide gel mobilities of Renilla luciferase-targeting and control siRNA-SF5 constructs. The loading amount of each sample was matched at 1.67 pmol. RNA was stained with SYBR Gold Nucleic Acid Gel Stain, which detects double-stranded nucleic acids more sensitively than single-stranded nucleic acids.[11] Arrowheads indicate the bands used for percent (%) yield calculations.

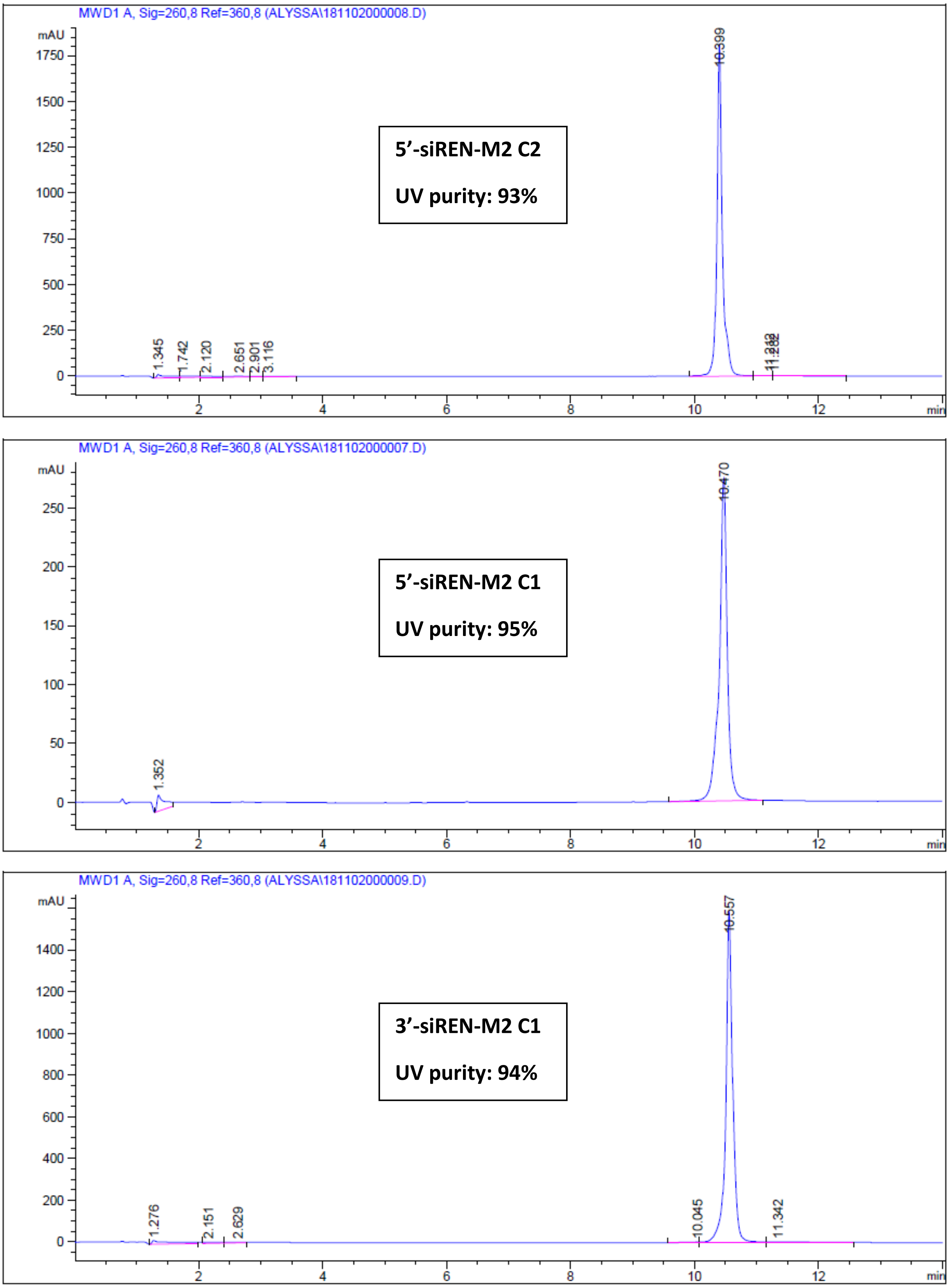

Figure S8. Chromatograms for selected oligoribonucleotides.

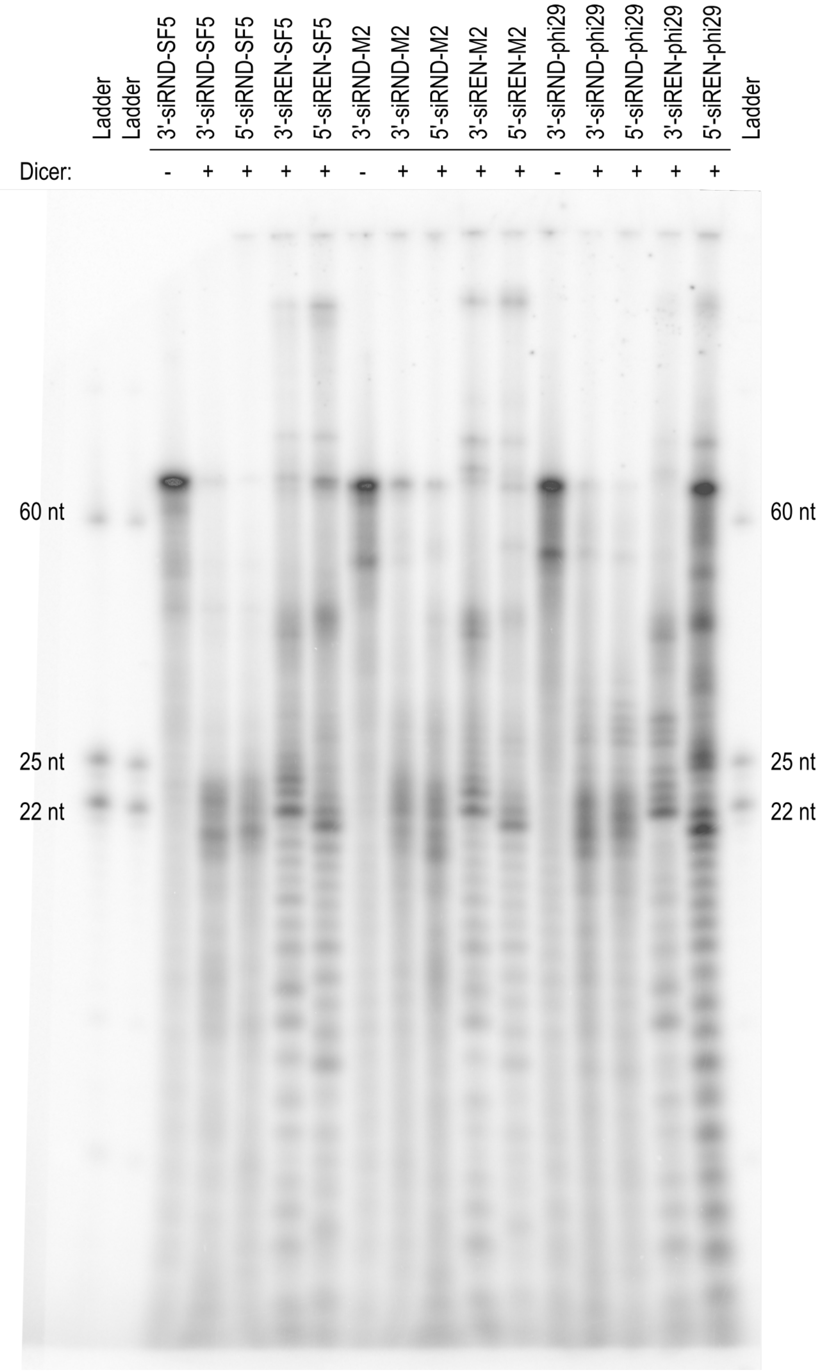

Figure S9. 20% denaturing gel mobilities of Renilla luciferase-targeting and control siRNA-3WJ constructs following incubation with (+) or without (-) recombinant human Dicer for 1 h at 37°C. The 25- and 22-nucleotide (nt) labels mark the expected range of Dicer cleavage products. The gel is representative of three independent replicates.

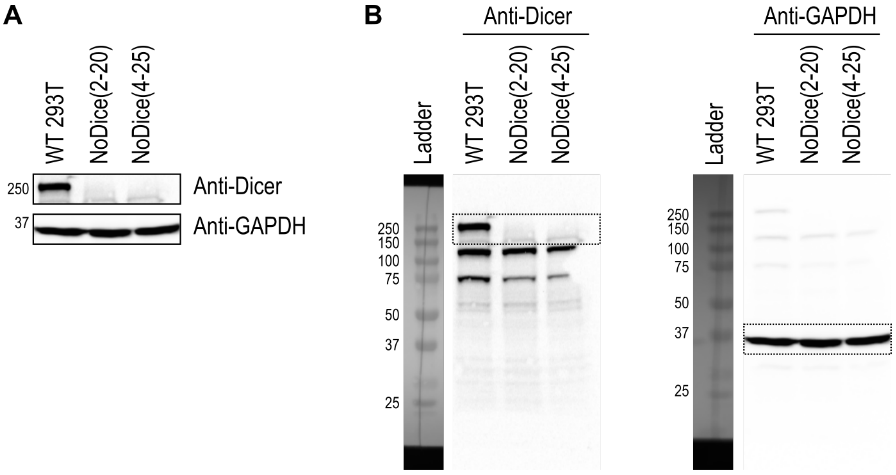

Figure S10. (A) Cropped western blots for WT 293T, NoDice(2-20) cells, and NoDice(4-25) cells.[9] (B) Full western blots for WT 293T, NoDice(2-20) cells, and NoDice(4-25) cells.[9]

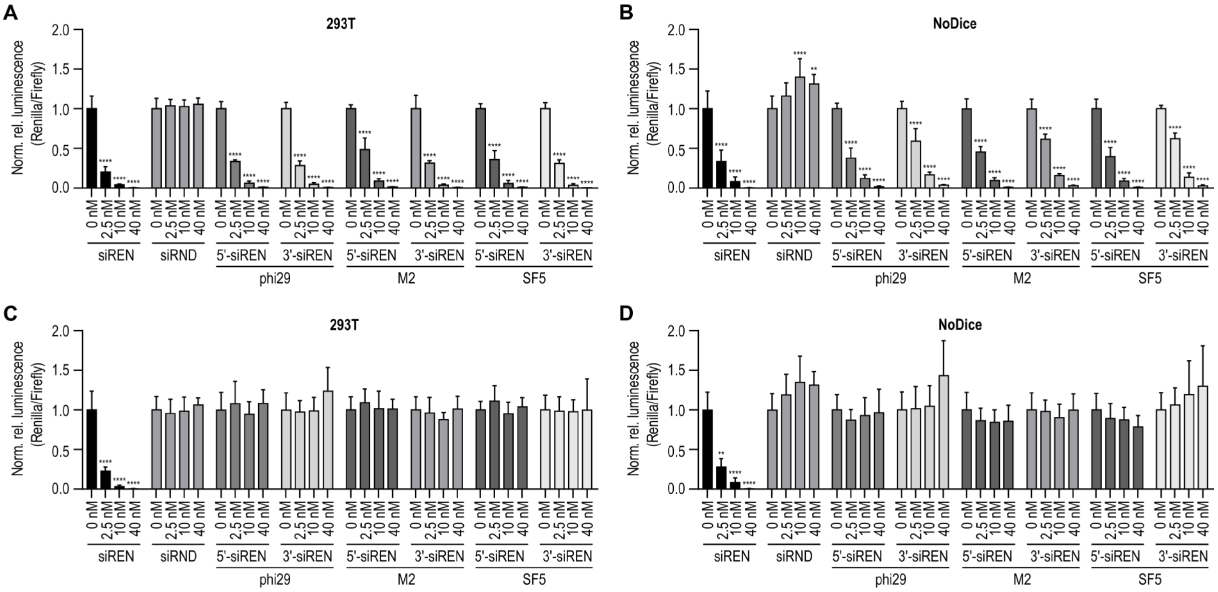

Figure S11. (A) Activity of Renilla luciferase-targeting siRNA-3WJs on a Renilla luciferase reporter in 293T cells. (B) Activity of Renilla luciferase-targeting siRNA-3WJs on the same reporter in Dicer-deficient (NoDice) cells.[9] (C) Activity of control siRNA-3WJs on a Renilla luciferase reporter in 293T cells. (D) Activity of control siRNA-3WJs on the same reporter in NoDice cells.[9] In (A) through (D), siREN and siRND served as positive and negative controls. Treatments were transfected at 0, 2.5, 10, and 40 nM. Data are mean Renilla/Firefly values normalized to the 0 nM treatment ± SD (n=3). Statistics are two-way ANOVA with Dunnett's multiple comparisons test against the 0 nM treatment: *P≤0.05, **P≤0.01, ***P≤0.001, ****P≤0.0001.

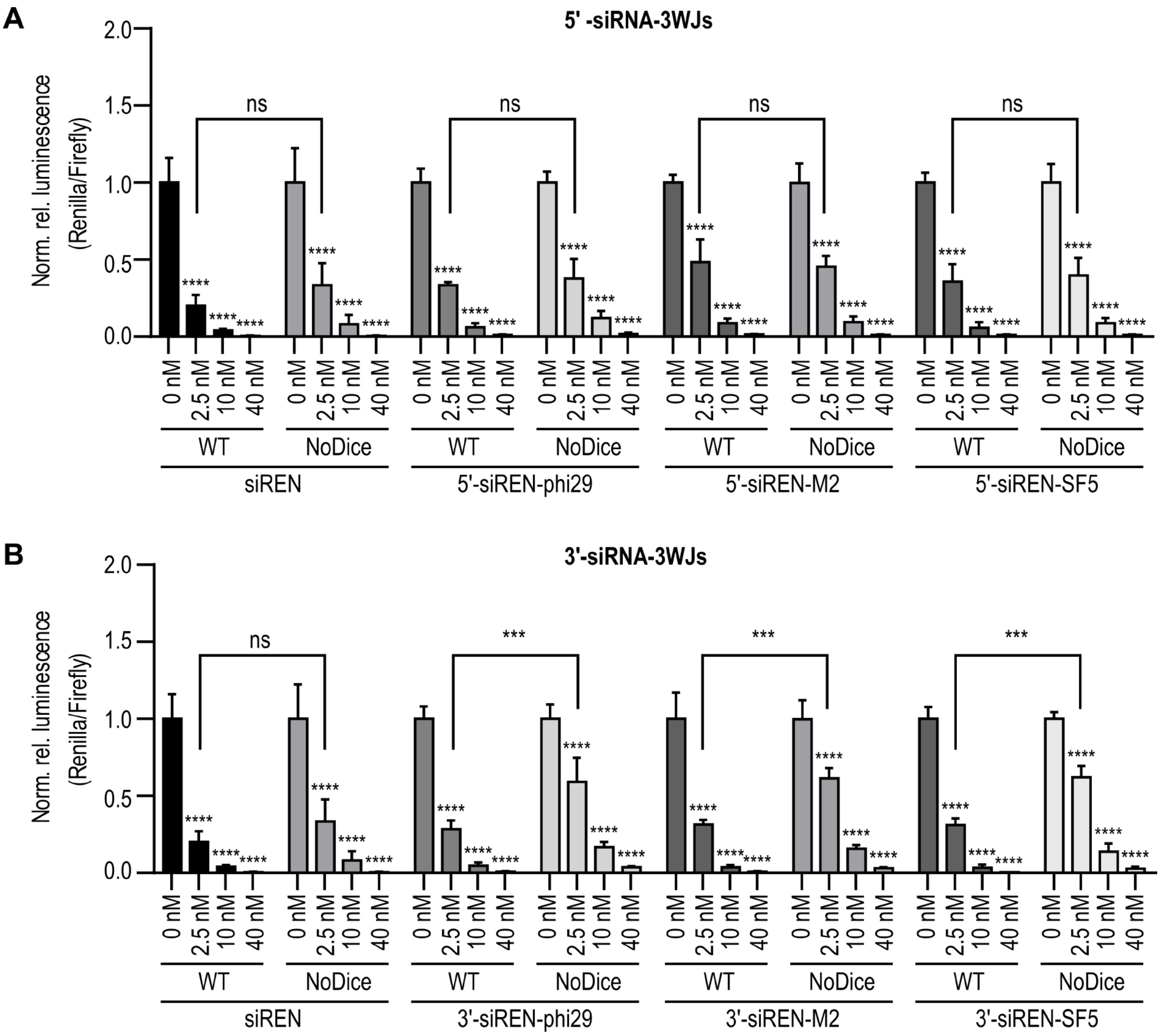

Figure S12. (A) Activity of Renilla luciferase-targeting 5'-siRNA-3WJs in wild-type (WT) versus Dicer-deficient (NoDice) cells.[9] (B) Activity of Renilla luciferase-targeting 3'-siRNA-3WJs in WT versus NoDice cells.[9] Treatments were transfected at 0, 2.5, 10, and 40 nM. Data are mean Renilla/Firefly values normalized to the 0 nM treatment ± SD (n=3). Statistics are two-way ANOVA with Dunnett's multiple comparisons test against the 0 nM treatment or Tukey's multiple comparisons test between 2.5 nM treatments: *P≤0.05, **P≤0.01, ***P≤0.001, ****P≤0.0001.

Table S5. Sequences of design-variant siRNA-M2 constructs. Antisense sequences are underlined. Adapters are bold.

Construct	Fusion helix	Strand	Sequence (5' to 3')
5'-siREN-M2-2	S1/S2	C1	UUUCUCGCCCUCUUCGCUCUUGCACAUGUCACGGGGUACGAAAACGUACCCUCUUACUA
	S1/S2	C2	UUGCAAAAGCAAUAGUAUGGCACAUGUGC**AA**GAGCGAAGAGGGCGAGAAAUU
3'-siREN-M2-2	S1/S2	C1	GAGCGAAGAGGGCGAGAAAUUGCACAUGUCACGGGGUACGAAAACGUACCCUCUUACUA
	S1/S2	C2	UUGCAAAAGCAAUAGUAUGGCACAUGUGC**AA**UUUCUCGCCCUCUUCGCUCUU
5'-siREN-M2-3	S2/S3	C1	UUUCUCGCCCUCUUCGCUCUUCGUACCCUCUUACUAUUGCAAAAGCAAUAGUAUGGCACAUG
	S2/S3	C2	UGCAAAAGCACAUGUCACGGGGUACG**AA**GAGCGAAGAGGGCGAGAAAUU
3'-siREN-M2-3	S2/S3	C1	GAGCGAAGAGGGCGAGAAAUUCGUACCCUCUUACUAUUGCAAAAGCAAUAGUAUGGCACAUG
	S2/S3	C2	UGCAAAAGCACAUGUCACGGGGUACG**AA**UUUCUCGCCCUCUUCGCUCUU

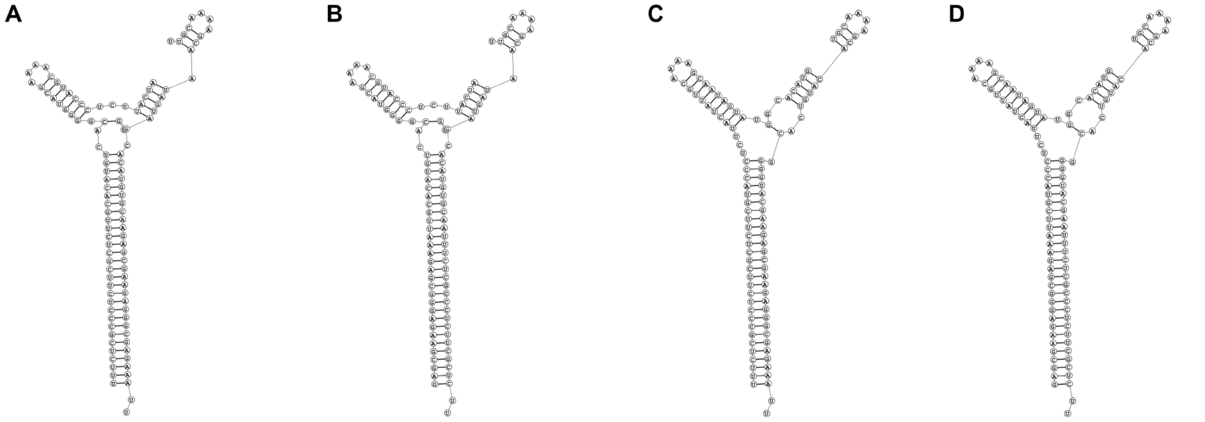

Figure S13. (A) Minimum free energy prediction for 5’-siREN-M2-2. (B) Minimum free energy prediction for 3’-siREN-M2-2. (C) Minimum free energy prediction for 5’-siREN-M2-3. (D) Minimum free energy prediction for 3’-siREN-M2-3. Predictions were made using the RNAstructure web servers version 6.0.1.[6] Results are bifold calculations on RNA using default parameters.

Table S6. Calculated and measured masses of design-variant siRNA-M2 construct component strands.

Construct	Fusion helix	Strand	Calculated mass (g/mol)	Measured mass (g/mol)
5'-siREN-M2-2	S1/S2	C1	18626.1	18626.88
	S1/S2	C2	16937.4	16936.72
3'-siREN-M2-2	S1/S2	C1	19090.6	19089.95
	S1/S2	C2	16472.9	16472.53
5'-siREN-M2-3	S2/S3	C1	19552.6	19553.81
	S2/S3	C2	16010.8	16010.47
3'-siREN-M2-3	S2/S3	C1	20017.2	20018.21
	S2/S3	C2	15546.3	15546.48

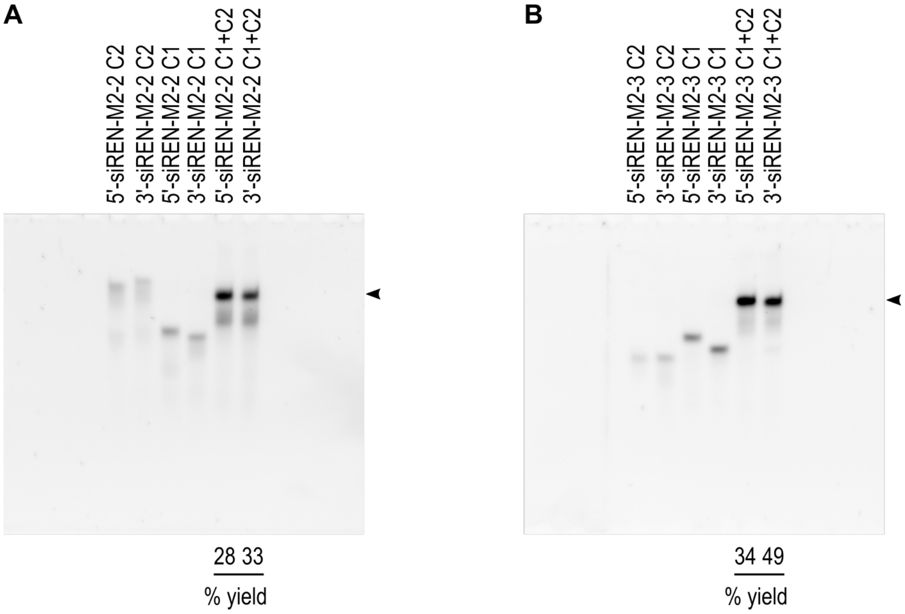

Figure S14. (A) 12% non-denaturing polyacrylamide gel mobilities of siRNA-M2-2 constructs. (B) 12% non-denaturing polyacrylamide gel mobilities of siRNA-M2-3 constructs. The loading amount of each sample was matched at 1.67 pmol. RNA was stained with SYBR Gold Nucleic Acid Gel Stain, which detects double-stranded nucleic acids more sensitively than single-stranded nucleic acids.[11] Arrowheads indicate the bands used for percent (%) yield calculations.

Table S7. Sequences of GFP-targeting and control siRNA-M2 constructs. Antisense sequences are underlined. Adapters are bold.

Construct	Fusion helix	Strand	Sequence (5' to 3')
5'-siGFP-M2	S3/S1	C1	UCCUUGAAGAAGAUGGUGCGCGCAAUAGUAUGGCACAUGUGCAAAAGCACAUGUCACGGGG
	S3/S1	C2	UAGGAAAACCUACCCUCUUACUAUUGC**GC**GCACCAUCUUCUUCAAGGACG
3'-siGFP-M2	S3/S1	C1	GCACCAUCUUCUUCAAGGACGGCAAUAGUAUGGCACAUGUGCAAAAGCACAUGUCACGGGG
	S3/S1	C2	UAGGAAAACCUACCCUCUUACUAUUGC**CG**UCCUUGAAGAAGAUGGUGCGC
5'-siPURO-M2	S3/S1	C1	GCGGUCCGGGUCGACGGUGUGGCAAUAGUAUGGCACAUGUGCAAAAGCACAUGUCACGGGG
	S3/S1	C2	UAGGAAAACCUACCCUCUUACUAUUGC**CA**CACCGUCGACCCGGACCGCCA
3'-siPURO-M2	S3/S1	C1	CACCGUCGACCCGGACCGCCAGCAAUAGUAUGGCACAUGUGCAAAAGCACAUGUCACGGGG
	S3/S1	C2	UAGGAAAACCUACCCUCUUACUAUUGC**UG**GCGGUCCGGGUCGACGGUGUG

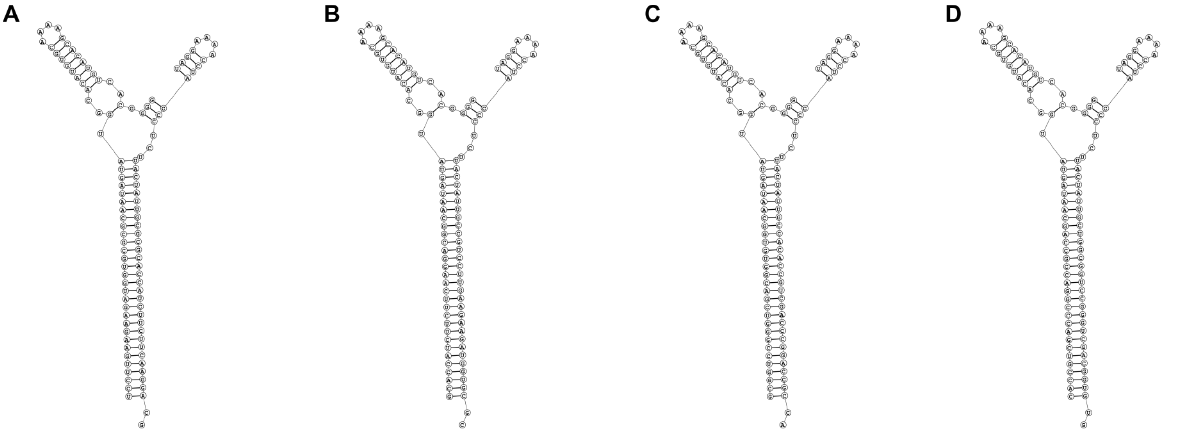

Figure S15. (A) Minimum free energy prediction for 5’-siGFP-M2. (B) Minimum free energy prediction for 3’-siGFP-M2. (C) Minimum free energy prediction for 5’-siPURO-M2. (D) Minimum free energy prediction for 3’-siPURO-M2. Predictions were made using the RNAstructure web servers version 6.0.1.[6] Results are bifold calculations on RNA using default parameters.

Table S8. Calculated and measured masses of GFP-targeting and control siRNA-M2 construct component strands.

Construct	Fusion helix	Strand	Calculated mass (g/mol)	Measured mass (g/mol)
5’-siGFP-M2	S3/S1	C1	19759.0	19757.90
	S3/S1	C2	15842.5	15841.21
3’-siGFP-M2	S3/S1	C1	19638.9	19642.45
	S3/S1	C2	15962.6	15963.02
5’-siPURO-M2	S3/S1	C1	19822.0	19822.90
	S3/S1	C2	15838.6	15838.13
3’-siPURO-M2	S3/S1	C1	19651.0	19652.61
	S3/S1	C2	16026.6	16026.16

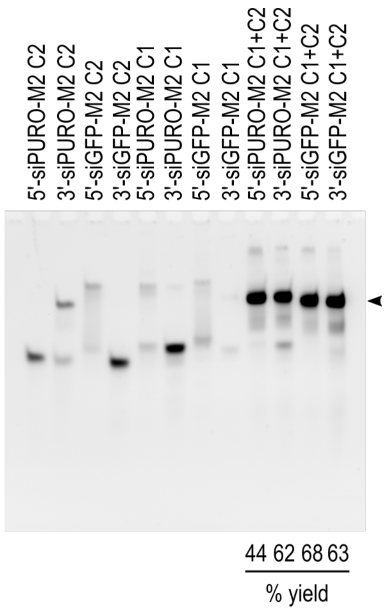

Figure S16. 12% non-denaturing polyacrylamide gel mobilities of GFP-targeting and control siRNA-M2 constructs. The loading amount of each sample was matched at 1.67 pmol. RNA was stained with SYBR Gold Nucleic Acid Gel Stain, which detects double-stranded nucleic acids more sensitively than single-stranded nucleic acids.[11] The arrowhead indicates the bands used for percent (%) yield calculations.

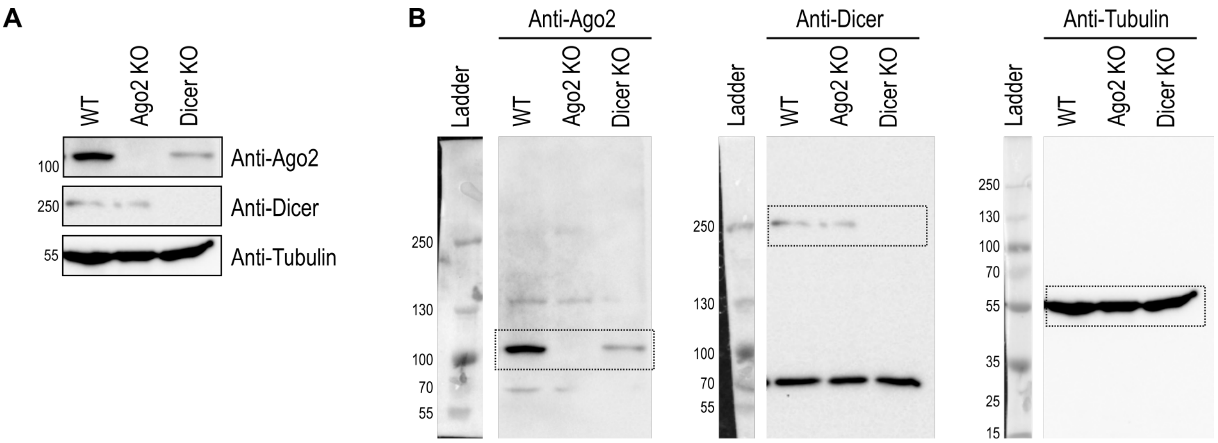

Figure S17. (A) Cropped western blots for WT, Ago2-KO, and Dicer-KO GFP-expressing 293T cells. (B) Full western blots for WT, Ago2-KO, and Dicer-KO GFP-expressing 293T cells.

Table S9. List of primers used: Guide cloning.

hsaDicer-G3-S	CACCGCCGCACAAAATGTTGGTAAA
hsaDicer-G3-AS	AAACTTTACCAACATTTTGTGCGGC
hsaAgo2-G3-S	CACCGGCCACCATGTACTCGGGAGC
hsaAgo2-G3-AS	AAACGCTCCCGAGTACATGGTGGCC

Table S10. List of primers used: T7E1 validation.

CRISPR validation oligos	
gDNA-hsaDicer-G3-S	TATTGGTAGTTCTGGGACCCT
gDNA-hsaDicer-G3-AS	AGCATGACGTATCAGCAATGA
gDNA-hsaAgo2-G3-S	GCGTCGGGTAAACCTGTTTGG
gDNA-hsaAgo2-G3-AS	ATGCGAGGACGCTGGGA

References

[1] A. C. Hill, S. J. Schroeder, *RNA* **2017**, *23*, 521-529.
[2] D. W. Binzel, E. F. Khisamutdinov, P. Guo, *Biochem.* **2014**, *53*, 2221-2231.
[3] S. Bailey, J. Wichitwechkarn, D. Johnson, B. E. Reilly, D. L. Anderson, J. W. Bodley, *J. Biol. Chem.* **1990**, *265*, 22365-22370.
[4] J. A. Zagalak, M. Menzi, F. Schmich, H. Jahns, A. M. Dogar, F. Wullschleger, H. Towbin, J. Hall, *RNA* **2015**, *21*, 2132-2142.
[5] Y. Shu, M. Cinier, S. R. Fox, N. Ben-Johnathan, P. Guo, *Mol. Ther.* **2011**, *19*, 1304-1311.
[6] J. S. Reuter, D. H. Mathews, *BMC Bioinform.* **2010**, *11*, 129.
[7] C. A. Schneider, W. S. Rasband, K. W. Eliceiri, *Nat. Methods* **2012**, *9*, 671-675.
[8] V. Schlösser, J. Hall, *Anal. Biochem.* **2019**, *579*, 35-37.
[9] H. P. Bogerd, A. W. Whisnant, E. M. Kennedy, O. Flores, B. R. Cullen, *RNA* **2014**, *20*, 923-937.
[10] N. E. Sanjana, O. Shalem, F. Zhang, *Nat. Methods* **2014**, *11*, 783-784.
[11] R. S. Tuma, M. P. Beaudet, X. Jin, L. J. Jones, C. Y. Cheung, S. Yue, V. L. Singer, *Anal. Biochem.* **1999**, *268*, 278-288.
